# A DFT Mechanistic Study on Base-Catalyzed Cleavage of the *β*-O-4 Ether Linkage in Lignin: Implications for Selective Lignin Depolymerization

**DOI:** 10.3389/fchem.2022.793759

**Published:** 2022-02-17

**Authors:** Mary Mensah, Richard Tia, Evans Adei, Nora H. de Leeuw

**Affiliations:** ^1^ Department of Chemistry, Kwame Nkrumah University of Science and Technology, Kumasi, Ghana; ^2^ School of Chemistry, Cardiff University, Cardiff, United Kingdom; ^3^ Department of Earth Sciences, Utrecht University, Utrecht, Netherlands; ^4^ School of Chemistry, University of Leeds, Leeds, United Kingdom

**Keywords:** lignin, depolymerization, aryl ether, *β*-O-4, lignin valorization

## Abstract

The detailed mechanism of the base-catalyzed C-C and C-O bond cleavage of a model compound representing the *β*-O-4 linkage in lignin is elucidated using DFT calculations at the M06/6-31G* level of theory. Two types of this linkage have been studied, a C2 type which contains no *γ*-carbinol group and a C3 type which contains a *γ*-carbinol. Cleavage of the C2 substrate is seen to proceed *via* a 6-membered transition structure involving the cation of the base, the hydroxide ion and the α-carbon adjacent to the ether bond. The reaction with KOH has the lowest activation barrier of 6.1 kcal mol^−1^ with a calculated rate constant of 2.1 × 10^8^ s^−1^. Cleavage of the C3 substrate is found to proceed *via* two pathways: an enol-formation pathway and an epoxide-formation pathway. The first path is the thermodynamically favored pathway which is similar to the pathway for the C2 substrate and is the preferred pathway for the isolation of an enol-containing monomer. The second path is the kinetically favored pathway, which proceeds *via* an 8-membered transition state involving a hydrogen hopping event, and is the preferred pathway for the isolation of an epoxide-containing monomer. The KOH-catalyzed reaction also has the lowest activation barrier of 10.1 kcal mol^−1^ along the first path and 3.9 kcal mol^−1^ along the second path, with calculated rate constants of 2.4 × 10^5^s^−1^ and 8.6 × 10^9^s^−1^ respectively. Overall, the results provide clarity on the mechanism for the base-catalyzed depolymerization of lignin to phenolic monomers. The results also suggest both NaOH and KOH to be the preferred catalysts for the cleavage of the *β*-O-4 linkage in lignin.

## Introduction

The feasibility of lignocellulosic biorefineries replacing petroleum and petrochemical refineries depends on conversion of both the cellulose fraction and lignin fraction of biomass to value-added products (Azadi et al., 2013). Over the years, valorization of the cellulose fraction for the production of biofuels and chemicals has been thoroughly studied and developed techniques have been employed industrially (Dutta et al., 2012; Ruppert et al., 2012; Akhtari et al., 2014). Lignin which accounts for 40% by energy in lignocellulose is still mostly a waste stream in the pulping and biorefinery processes and only 5% of lignin is used in low-value commercial applications (Hu et al., 2011). Due to the complex nature of lignin, having strong carbon-carbon bonds and ether bonds holding its three major monomeric units together, there is still a general lack of efficient processes for the utilization of lignin (Parthasarathi et al., 2011; Elder, 2014). One major benefit that lignin valorization holds is the production of 100% biomass-derived jet fuel because lignin is the only abundant renewable source of aromatic hydrocarbons in nature (Chakar and Ragauskas, 2004; Hileman and Stratton, 2014) and aromatics play a vital role in jet fuel quality and safety, specifically the fuel’s elastomeric swelling, material compatibility and lubricity characteristics (Huber et al., 2006).

**SCHEME 1:**
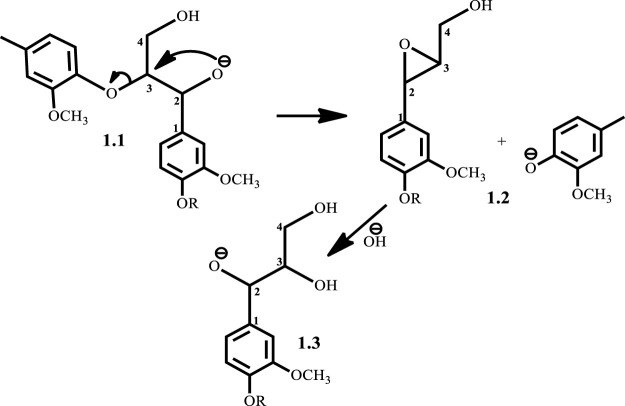
Mcdonough’s (Lavoie et al., 2011) proposed mechanism for base catalyzed depolymerization of lignin.

**SCHEME 2:**
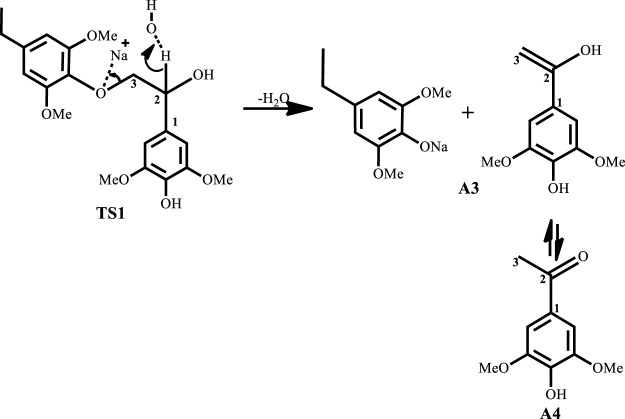
Roberts et al. (Roberts et al., 2010) proposed mechanism for base catalyzed depolymerization of lignin.

**SCHEME 3:**
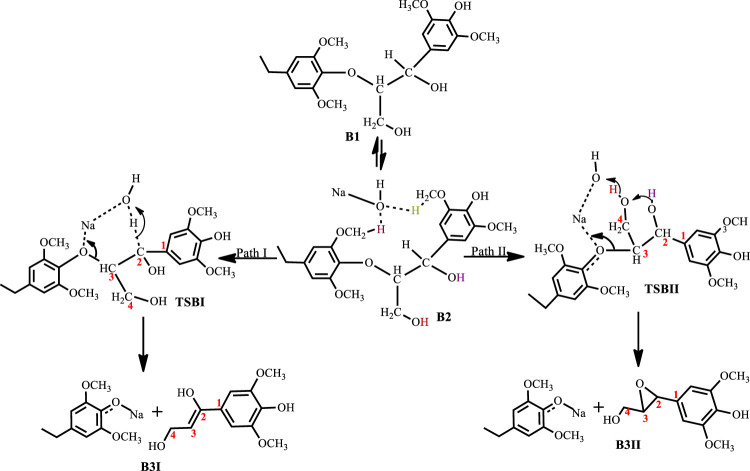
Mechanism for the base catalyzed cleavage of a lignin *β*-O-4 ether bond containing a *γ*-glycol.

With significant research still ongoing to convert lignin into value-added products, three general routes exist for lignin conversion. The first is the production of oil through pyrolysis, the second is the conversion to syngas (Huber et al., 2006; Pandey and Kim, 2011; Lavoie et al., 2011), while the third is chemical treatment to produce target molecules. Current research focuses on the third route as the first two typically demand severe conditions, while the third is relatively mild and is advantageous for both reaction control and high selectivity. One such mild treatment is the use of sodium hydroxide for the treatment of lignin in aqueous solution at temperatures of about 300°C from which phenols and phenol derivatives are obtained (Huber et al., 2006). This process was formerly used to delignify paper making in the pulping industry, as lignin is an impediment to the paper making process (Mcdonough, 1993). In 2011, Roberts et al. (Roberts et al., 2011a) carried out NaOH-catalyzed treatment of lignin in water to give syringol and its derivatives as major products. Toledano et al. (Toledano et al., 2012) screened various base catalysts, including KOH and LiOH, for the depolymerization of lignin from olive tree pruning in aqueous solution where they reported that the nature of the base governs monomer yield. Hartwig et al. (Sergeev and Hartwig, 2011) also reported the cleavage of the *β*-O-4 lignin model compound in the presence of NaO^*t*^Bu alone when used as a co-catalyst, yielding guaiacol at 89% yield. One major disadvantage of base-catalyzed depolymerization of lignin is the repolymerization reactions which occur during the process, thereby limiting monomer yields. Lercher et al.(Roberts et al., 2011a) reported the success of boric acid as an excellent capping agent in hindering secondary reactions and improving monomer yields. Phenol was reported to be a better capping agent by Toledano et al. (Toledano et al., 2014) since boric acid allowed for more char formation in the final product, while phenol yielded low char content and decreased residual lignin down to 25% (Toledano et al., 2014). It is said that during the alkaline delignification process, cleavage of *β*-O-4 ether bond is the dominant reaction and according to Mcdonough et al. (Lavoie et al., 2011) the reaction takes place as a result of deprotonated hydroxyl groups on the α-carbon that act as nucleophiles in displacing the neighboring aroxy substituent with the formation of an oxirane ring which is subsequently opened by addition of a hydroxide ion to form a glycol (Scheme 1). A more recent mechanism proposed by Roberts et al. (Roberts et al., 2010) postulates that the cleavage of the *β*-O-4 ether bond takes place heterolytically *via* a six-membered transition state in which the sodium ion and hydroxide ion participate (Scheme 2). The sodium cation forms a cation adduct with the lignin, thereby polarizing the ether bond which eventually cleaves to form a sodium phenolate intermediate and 4-hydroxylvinyl syringol. A third mechanism results from this study (Scheme 3), wherein cleavage of the *β*-O-4 ether bond which contains the *γ-*carbinol could proceed *via* two pathways; this mechanism is discussed in detail in *Results and Discussions Section*.

Despite the progress that has been made in the area, there is the need for better understanding of the molecular-level processes of this base-catalyzed depolymerization reactions to delineate what happens at the molecular level. This will help fine-tune reaction selectivity towards higher monomer yields as well as improve energy efficiency for industrial application (Kleine et al., 2013; Toledano et al., 2014).

**FIGURE 1:**
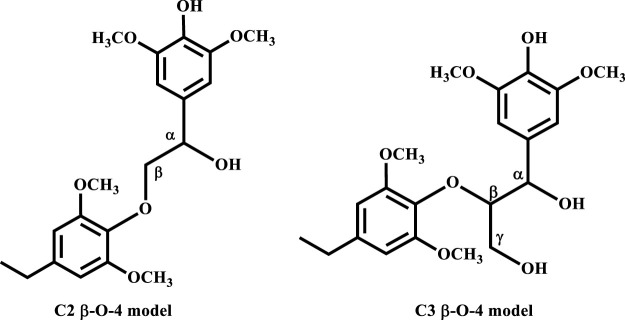
The two types of *β*-O-4 linkage.

Here, we have employed calculations based on the density functional theory (DFT) to carry out an exploratory/predictive molecular modelling study to provide molecular-level insight into the details of the base-catalyzed cleavage of the C-O bond in a *β*-O-4 linkage, which is the predominant linkage found in lignin (Kleine et al., 2013). The *β*-O-4 substrate chosen for this study would show the formation of syringol derivatives which is reported to constitute the largest fraction of monomer products obtained from the base catalyzed depolymerization of lignin (Mcdonough, 1993; Roberts et al., 2011a). The energetics (thermodynamics and kinetics) of the elementary steps for the catalyzed cleavage of a C2 *β*-O-4 linkage as well as a C3 *β*-O-4 linkage using NaOH, KOH, LiOH and NaO^*t*^Bu were studied in both the gas phase and the solvated phase following the two mechanisms proposed in Schemes 1, 2. The C2 *β*-O-4 linkage is a simpler model substrate typically used in both experimental and computational studies, while the C3 *β*-O-4 linkage better represents the linkage in the actual lignin framework (Figure 1).

## Computational Details

All calculations were carried out using the Spartan ‘14 Molecular Modelling program (Spartan, 2011) and Gaussian 09’ package (Frisch et al., 2016). The structures and energies of all the stationary points involved along the reaction pathway were computed at the M06/6-31G* level of theory. The M06 functional is a hybrid meta-gradient-corrected functional (*meta*-GGA) with 27% of Hartree-Fock exchange. Zhao et al. have carried out a comprehensive benchmarking study to evaluate the accuracy of this method against a number of properties, including barrier heights, bond dissociation energies, and non-covalent interactions (Zhao and Truhlar, 2008). The parameters considered in their study are the focus of the present work and we have therefore chosen the same method as recommended for the study of organometallic thermochemistry, as well as non-covalent interactions (Zhao and Truhlar, 2008). The 6-31G* basis set is a double-zeta basis set, known to give reliable results for charge-localized anions such as the hydroxide (Rassolov et al., 1998). All stationary points were characterized by full frequency calculations. Minima (reactants, intermediates and products) were shown to have a Hessian matrix whose eigenvalues are all positive, leading to vibrational frequencies which are real, while transition states were shown to have a Hessian matrix having all positive eigenvalues except a single negative eigenvalue characterized by a vibration along the reaction coordinate. In Supplementary Table S1 of the Supplementary Information, we have provided a comparison of the absolute electronic energies and relative energies computed using both 6-31G* and 6-311G* basis sets and a comparison of optimized geometrical parameters at the double and triple zeta basis sets. The 6-311G* optimized geometries reproduce the 6-31G* structures and the calculated relative energies using the two methods are within 3 kcal mol^−1^, thus providing confidence that the use of the 6-31G* basis set is suitable for this work.

Input structures for transition state optimizations were obtained after a potential energy surface (PES) scan was performed for each reaction step. This calculation was also useful in showing that the minima obtained do in fact correspond to the reactant and product for that step. The PES scan gives an approximate transition state structure which is then submitted for a transition state calculation using the synchronous Transit-Guided Quasi-Newton (STQN) Method developed by Schlegel and coworkers (Peng and Bernhard Schlegel, 1993). General effects of a surrounding solvent on the computed geometries and energies of the stationary points along the reaction pathway were evaluated using a polarizable continuum model derived with an integral equation formalism (IEF-PCM) (Mennucci et al., 1997) of water solvation, which is the solvent used in experiments. The PCM approach is widely used for this type of computational study and our choice of solvent model was guided by detailed benchmarking studies (Miguel et al., 2016), which indicate that the model should provide reliable energetic trends in reaction pathways. All reported energies are Gibbs free energies.

## Results and Discussions

### Base Catalyzed Cleavage of C2 *β*-O-4 Substrate

**FIGURE 2:**
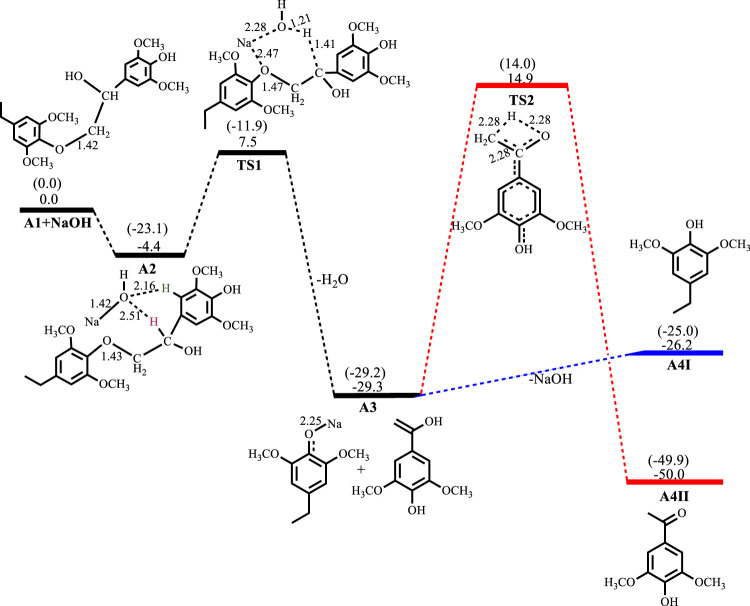
Free energy profile for NaOH catalyzed cleavage of C2 *β*-O-4 substrate computed at M06/6-31G* level of theory. Relative energies in kcal mol^−1^. Gas-phase energies in parenthesis and selected bond distances in Å. (XYZ coordinates, Supplementary Table S2, Supporting Information).

**FIGURE 3:**
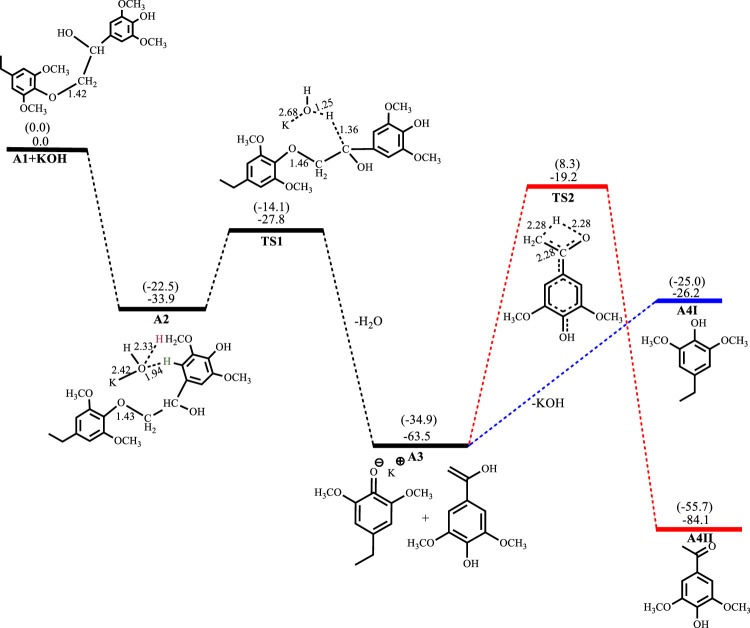
Free energy profile for KOH catalyzed cleavage of C2 *β*-O-4 substrate. Relative energies in kcal mol^−1^ computed at M06/6-31G* level of theory. Gas-phase energies in parenthesis and selected bond distances in Å. (XYZ coordinates, Supplementary Table S3, Supporting Information).

**FIGURE 4:**
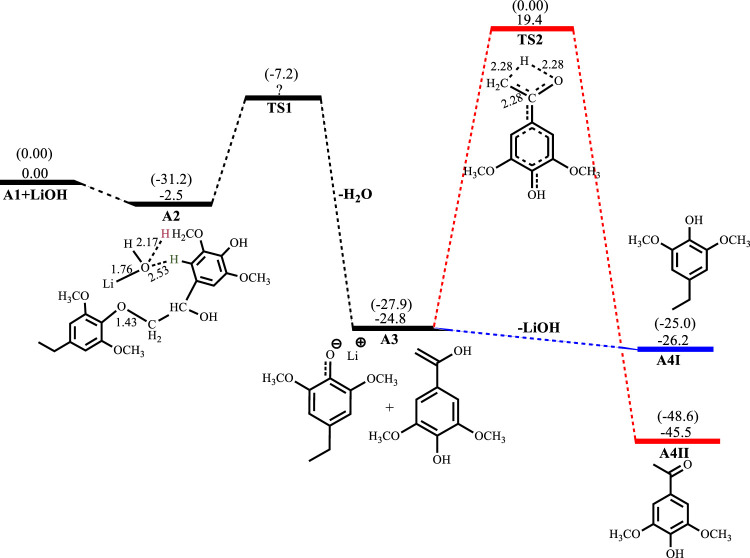
Free energy profile for LiOH catalyzed cleavage of C2 *β*-O-4 substrate. Relative energies in kcal mol^−1^ computed at M06/6-31G* level of theory. Gas-phase energies in parenthesis and selected bond distances in Å. (XYZ coordinates, Supplementary Table S4, Supporting Information).

**FIGURE 5:**
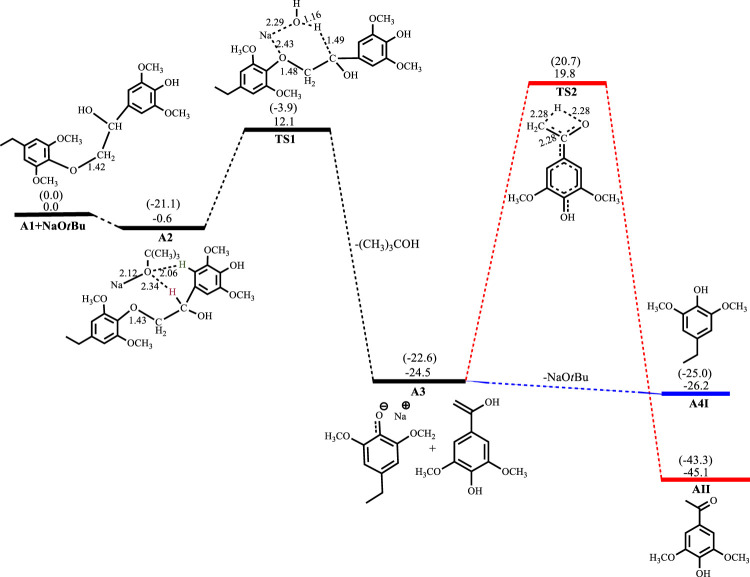
Free energy profile for NaO^*t*^Bu catalyzed cleavage of C2 *β*-O-4 substrate. Relative energies in kcal mol^−1^ computed at M06/6-31G* level of theory. Gas-phase energies in parenthesis and selected bond distances in Å. (XYZ coordinates, Supplementary Table S5, Supporting Information).

Figures 2–5 show the optimized geometries and relative energies of the stationary points for the cleavage of the C2 substrate using NaOH, KOH, LiOH and NaO^t^Bu, as well as the transformation of the cleaved products to the final products 4-ethylsyringol and methoxy-4-hydroxyacetophenone. The catalytic cleavage is exergonic for all bases used (ΔG^NaOH^ = −24.9 kcal mol^−1^, ΔG^KOH^ = −29.6 kcal mol^−1^, ΔG^LiOH^ = −22.3 kcal mol^−1^, ΔG^NaOtBu^ = −23.9 kcal mol^−1^) although it was determined that an intermediate (**A2**) is formed before the cleavage occurs. The intermediate shows electrostatic interactions between the hydroxide ion and two of the hydrogens on the *β*-O-4 substrate which is assumed to stabilize the intermediate. These electrostatic interactions are found to be stronger in the gas phase than in the solvated phase (A2^NaOH^; O---H_gas_ = 1.96Å and 2.08Ǻ, O---H_solv_ = 2.51Å and 2.16Å) which could explain why **A2** is more stable in the gas phase for all the bases except for KOH, where **A2** ^KOH^ is found to be more stable in the solvated phase than in the gas phase. The catalytic C-O bond cleavage is shown to proceed *via* the 6-membered transition state **TS1** where the hydroxide ion deprotonates the α-carbon to form water and the C-O bond is cleaved (Scheme 2). The KOH-catalyzed reaction has the lowest activation barrier of 6.1 kcal mol^−1^, compared to NaOH and NaO^*t*^Bu which have activation barriers of 11.9 and 12.8 kcal mol^−1^, respectively. The transition state for the LiOH process could only be located in the gas phase. These findings show that, if the reactions are kinetically controlled, the KOH catalyst is the most efficacious for the catalytic cleavage of the C2 *β*-O-4 ether bond which in turn means that stronger bases facilitate faster cleavage. The NaOH is said to be recovered from the reaction by dissolving the organic fraction in acetone (Roberts et al., 2011a) and although the mechanism for this step is not explored, it is assumed that the hydrogen source for the conversion of the phenolate monomer to its phenol motif **A4I** observed in experiment comes from this step. The phenol monomer **A4I** was found to be much less stable than the acetophenone monomer **A4II**, which suggests that the phenolate monomer could be the most likely precursor for any repolymerization reaction taking place and the KOH-catalyzed system would undergo the highest amount of repolymerization (Toledano et al., 2012) as it forms the least stable phenol motif at ΔG(A4I)^KOH^ = 37.3 kcal mol^−1^. The acetophenone monomer **A4II** was formed *via* rearrangement of the hydroxyvinyl-syringol in **A3** and was found to be the thermodynamically preferred product. This is expected since the equilibrium between an enol and a ketone lies far toward the keto form. It is, however, unlikely that the rearrangement would go *via* **TS2** since the activation barrier is very high (*E*a = 44.2 kcal mol^−1^). The activation barrier and formation energy obtained for all base-catalyzed reactions is the same for the formation of **A4II**.

In the reaction of NaOH with the C2 *β*-O-4 substrate, the intermediate **A2** ^**NaOH**^ is exergonic by −4.4 kcal mol^−1^. Catalytic C-O bond cleavage proceeds with an activation barrier of 11.9 kcal mol^−1^ and formation of the sodium phenolate and hydroxyvinyl-syringol intermediates **A3** ^**NaOH**^ is exergonic by −24.9 kcal mol^−1^.

Conversion of the phenolate in **A3** ^**NaOH**^ to the phenol **A4I** ^**NaOH**^ is endergonic by 3.1 kcal mol^−1^, while the rearrangement of the hydroxyvinyl-syringol into the more stable 3,5-dimethoxy-4-hydroxyacetophenone **A4II** ^**NaOH**^ has an activation barrier of 44.2 kcal mol^−1^ and is exergonic by 20.7 kcal mol^−1^, as shown in Figure 2. For the reaction involving KOH, **A2** ^**KOH**^ is exergonic by 33.9 kcal mol^−1^ while C-O bond cleavage is exergonic by 29.6 kcal mol^−1^ and needs to overcome an activation barrier of 6.1 kcal mol^−1^. Formation of **A4I** ^**KOH**^ is endergonic by 37.3 kcal mol^−1^ while the formation of **A4II** ^**KOH**^ has an activation barrier of 44.3 kcal mol^−1^ and is exergonic by 20.6 kcal mol^−1^, as shown in Figure 3.

The LiOH-catalyzed reaction shows the formation of **A2** ^**LiOH**^ to be exergonic by 2.5 kcal mol^−1^. the TS for the C-O bond cleavage has not been obtained in the solvated phase, but the C-O cleavage is exergonic by 22.3 kcal mol^−1^. Formation of **A4I** ^**LiOH**^ is slightly exergonic by 1.4 kcal mol^−1^, while the formation of **A4II** ^**LiOH**^ has an activation barrier of 44.2 kcal mol^−1^ and is exergonic by 20.7 kcal mol^−1^, as shown in Figure 4. In the reaction with NaO^*t*^Bu, the formation of **A2** ^**NaO*t*Bu**^ is almost thermos-neutral having a reaction energy of −0.6 kcal mol^−1^, whilst C-O bond cleavage proceeds with an activation barrier of 12.7 kcal mol^−1^ and is exergonic 23.9 kcal mol^−1^. Formation of **A4I** ^**NaO*t*Bu**^ is slightly exergonic by 1.7 kcal mol^−1^, while **A4II** ^**NaO*t*Bu**^ has an activation barrier of 44.3 kcal mol^−1^ and is found to be exergonic by 20.6 kcal mol^−1^, as shown in Figure 5.

### Base-Catalyzed Cleavage of C3 *β*-O-4 Substrate

**FIGURE 6:**
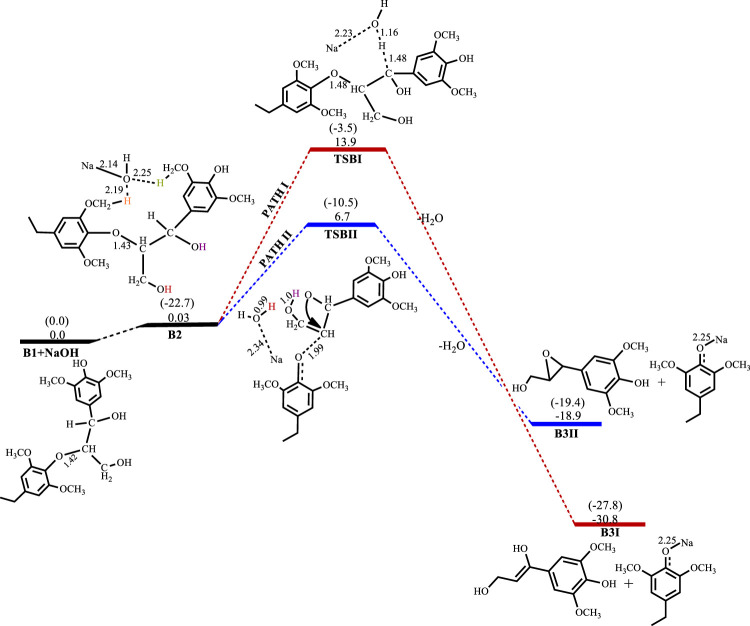
Free energy profile for NaOH catalyzed cleavage of C3 *β*-O-4 substrate. Relative energies in kcal mol^−1^ computed at M06/6-31G* level of theory. Gas-phase energies in parenthesis and selected bond distances in Å. (XYZ coordinates, Supplementary Table S6, Supporting Information).

The base-catalyzed cleavage of the C3 *β*-O-4 substrate also shows the presence of an intermediate, as was observed in the C2 cleavage reaction, although the solvated phase calculations show the intermediate **B2** to be quite unstable ΔG(B2)^NaOH^ = 0.03 kcal mol^−1^, ΔG(B2)^KOH^ = −1.8 kcal mol^−1^, ΔG(B2)^LiOH^ = 1.3 kcal mol^−1^, ΔG(B2)^NaOtBu^ = 1.9 kcal mol^−1^, where for the NaOH-catalyzed reaction the formation of the intermediate is almost thermos-neutral, while the NaO^*t*^Bu-catalyzed reaction gives the least stable intermediate and KOH gives the most stable intermediate. The catalytic cleavage is found to be an exergonic process that proceeds *via* two pathways (Scheme 3). The first pathway (Path I) involves a 6-membered transition state **TSBI** similar to that seen for the C2 substrate cleavage, wherein the hydroxide ion deprotonates the α-carbon which promotes C-O bond cleavage. The second pathway (Path II) shows an 8-membered transition state which involves the C-O bond cleavage accompanied by a hydrogen hop from the oxygen on the *γ*-carbon to the base hydroxide, followed by another hydrogen hop from the oxygen on the *α*-carbon to the deprotonated oxygen on the *γ*-carbon, resulting in the formation of an oxirane ring. This mechanism could be said to be akin to the Grotthuss mechanism, (Agmon, 1995) albeit, that the subject has been discussed in respect of hydroxonium ions or a network of water molecules (Roberts et al., 2011b; Chen et al., 2013). The observed mechanism for the proton transfer is similar to that discussed by Tuckerman et al. (Tuckerman et al., 2002) and it involves an initial reorientation of the coordinated complex **B2** (Scheme 3), whereas the movement of the hydroxyl ion of the base is accompanied by a hyper-coordination of the hydroxide ion on the *γ*-carbon, while the hydroxide ion on the *α*-carbon provides the incoming proton which replaces the proton transferred to the hydroxyl ion. Figure 6 shows the optimized geometries and relative energies of the stationary points for the cleavage of the C3 substrate using NaOH. Path I is found to be the thermodynamically favored pathway, having an activation barrier of 13.9 kcal mol^−1^ and a reaction energy of −30.8 kcal mol^−1^, while path II is the kinetically favored pathway having an activation barrier 6.7 kcal mol^−1^ and a reaction energy of −18.9 kcal mol^−1^.

Table 1 shows the relative energies obtained for the use of KOH, LiOH and NaO^*t*^Bu as catalysts for the cleavage of the C3 *β*-O-4 substrate. Reaction with KOH has the lowest activation barrier along both pathways (Path I and Path II) at E_a_^KOH^ = 10.1 and 3.9 kcal mol^−1^, respectively while C-O bond cleavage is exergonic by 33.0 and 21.1 kcal mol^−1^. Along Path I, the LiOH-catalyzed reaction has an activation barrier of 20.7 kcal mol^−1^ and a reaction energy of −27.6 kcal mol^−1^, while the NaO^*t*^Bu-catalyzed reaction has an activation barrier of 16.4 and a reaction energy of −27.8 kcal mol^−1^. Along Path II, the LiOH-catalyzed reaction has an activation barrier of 11.4 kcal mol^−1^ and a reaction energy of −15.7 kcal mol^−1^ while the NaO^*t*^Bu-catalyzed reaction has an activation barrier of 11.3 kcal mol^−1^ and a reaction energy of −15.9 kcal mol^−1^. This shows that along path I, the reaction involving NaOH is kinetically favored over the NaO^*t*^Bu-catalyzed reaction by 2.5 kcal mol^−1^ and over the LiOH-catalyzed reaction by 6.8 kcal mol^−1^. However, along path II the NaOH-catalyzed reaction is kinetically favored over both the LiOH-catalyzed and the NaO^*t*^Bu-catalyzed reaction by 4.7 and 4.6 kcal mol^−1^, respectively. This shows LiOH and NaO^*t*^Bu to have comparable catalytic activity for the cleavage of the C3 *β*-O-4 ether linkage found in lignin.

**TABLE 1:** Relative energies computed at M06/6-31G* level of theory for the catalyzed cleavage of the C3 *β*-O-4 substrate using KOH, LiOH and NaO^*t*^Bu. All energies are measured in kcal mol^−1^.

Base	*E*_*a*_(TSBI)	*E*_*a*_(TSBII)	*E*_f_(B3I)	*E*_f_(B3II)
KOH	10.1	3.9	−33.0	−21.1
LiOH	20.7	11.4	−27.6	−15.7
NaO^*t*^Bu	16.4	11.3	−27.8	−15.9

### Rate Constants for the Cleavage Reactions

The rate constants for all base-catalyzed cleavage reactions were calculated using the equation derived from the transition state theory (McQuarrie and Simon, 1997):

k(T)= KBThc°e−Δ‡G∘RT (1)

Where c*˚* is the standard concentration taken as 1, *T* = 298.15 K and Δ^*ǂ*^G˚ is the free energy of activation obtained for the reactions. The computed rate constants for all three base-catalyzed reactions studied are shown in Table 2.

**TABLE 2:** Rate constants (in s^−1^) for the catalyzed cleavage of the C-O bond in a C2 and C3 *β*- O-4 linkage using NaOH, KOH, LiOH and NaO^*t*^Bu.

Base catalyst	C2 cleavage/S^−1^	C3 cleavage(path I)/S^−1^	C3 cleavage(path II)/S^−1^
NaOH	1.2 × 10^4^	4.0 × 10^2^	7.6 × 10^7^
KOH	2.1 × 10^8^	2.4 × 10^5^	8.6 × 10^9^
LiOH	1.6 × 10^−5^ ^a^	4.2 × 10^−3^	2.7 × 10^4^
NaO^*t*^Bu	3.0 × 10^3^	5.9 × 10°	3.2 × 10^4^

a Gas phase activation energy used since TS1 for LiOH, catalyzed cleavage of the C2-substrate could only be obtained in the gas phase.

As expected, the KOH-catalyzed reaction proceeds much faster along all pathways and for both types of *β*-O-4 linkage studied.

## Conclusion

This study has shown that the base-catalyzed cleavage of the *β*-O-4 ether linkage in lignin begins with the formation of an intermediate, which is stabilized owing to electrostatic interactions between the hydroxide ion of the base and hydrogens on the phenyl ring adjacent to the ether bond. The transition state for the C-O bond cleavage in the *β*-O-4 linkage which does not contain a *γ*-carbinol (C2-substrate) involves a 6-membered transition state in which the hydroxide ion deprotonates the *α*-carbon adjacent to the ether bond and C-O bond cleavage occurs. In contrast, the cleavage of the *β*-O-4 linkage containing a *γ*-carbinol (C3-substrate) can proceed *via* two pathways: a thermodynamically favored pathway involving a TS similar to that of the C2-substrate and a kinetically favored pathway involving a unique 8-membered TS in which the C-O cleavage is accompanied by a hydrogen hopping event which could be akin to the Grotthuss mechanism. The thermodynamically favored pathway is also the preferred pathway for the isolation of the enol-containing monomer, while the kinetically favored pathway is the preferred pathway for the isolation of the epoxide-containing monomer. For the cleavage of the C2 substrate, the order of activation barriers with respect to the bases studied is LiOH > NaO^*t*^Bu > NaOH > KOH. The same order is observed along path I for the cleavage of the C3 substrate, while along path II the order of the activation energy barrier is LiOH = NaO^*t*^Bu > NaOH > KOH. The rate constants have also been calculated using the activation energies obtained, which show that the stronger base KOH rapidly promotes the C-O bond cleavage, in agreement with reports from experiment. These conclusions provide clarity on the mechanism of the base-catalyzed depolymerization of lignin to form phenolic monomers and suggest KOH and NaOH to be the preferred catalysts for the C-O bond cleavage in the *β*-O-4 linkage found in lignin.

## Data Availability

The original contributions presented in the study are included in the article/Supplementary Material, further inquiries can be directed to the corresponding author.
