# Supplementary material for: A DFT Mechanistic Study on Base-Catalyzed Cleavage of the β-O-4 Ether Linkage in Lignin: Implications for Selective Lignin Depolymerization
Source: Front Chem. 2022 Feb 17;10:793759. doi: 10.3389/fchem.2022.793759 (PMC8892242; doi:10.3389/fchem.2022.793759)
Supplement: Supplementary file 1 [file DataSheet1.docx]

**Supporting Information**

**A DFT Mechanistic Study on Base-Catalyzed Cleavage of the β-O-4 Ether Linkage in Lignin: Implications for Selective Lignin Depolymerization**

Mary Mensah^1^, Richard Tia^1^, Evans Adei^1^, Nora de Leeuw^2,3^

*^1^Department of Chemistry, Kwame Nkrumah University of Science and Technology, Kumasi, Ghana*

*^2^School of Chemistry, Cardiff University, Main Building, Park PI, Cardiff CF10 3AT, United Kingdom*

*^3^Department of Earth Sciences, Utrecht University, Princetonlaan 8a, 3584 CB Utrecht, The Netherlands*

**TABLE OF CONTENTS**

1. Preliminary studies of the reaction between Na^+^ and OH^-^ ions and the C2 substrate
2. Table S1. Benchmarking study: Electronic energies and relative energies of 6-31G* and 6-311G* optimized geometries of the parent reaction between the C2 substrate **A1** and NaOH.
3. Table S2. The Cartesian coordinates, total energy, zero-point vibrational energy and number of imaginary frequencies, of the optimized structures in Figure 2.
4. Table S3**.** The Cartesian coordinates, total energy, zero-point vibrational energy and number of imaginary frequencies, of the optimized structures in Figure 3.
5. Table S4**.** The Cartesian coordinates, total energy, zero-point vibrational energy and number of imaginary frequencies, of the optimized structures in Figure 4.
6. Table S5**.** The Cartesian coordinates, total energy, zero-point vibrational energy and number of imaginary frequencies, of the optimized structures in Figure 5.
7. Table S6**.** The Cartesian coordinates, total energy, zero-point vibrational energy and number of imaginary frequencies, of the optimized structures in Figure 6.

To account for the Arrhenius nature of NaOH, a pathway in which the cleavage reaction begins with the dissociation of the base and the OH ions get hydrolyzed in solution while the Na ion goes on to protonate the ether oxygen of the β-O-4 substrate forming a cation adduct and subsequent C-O cleavage occurs via the dehydrogenation of the Cα by the ether oxygen was considered. Figure S1 shows the relative energies and optimized geometries of the stationary points involved in the cationic cleavage of a C2 β-O-4 lignin substrate both in the gas phase and using a polariable continuum model. Formation of the cation adduct is highly endergonic by 127.05 kcalmol^-1^ in the gas phase and endergonic by 27.69 kcalmol^-1^ in the solvated phase . The C-O bond cleavage which occurs in a concerted fashion with the dehydrogenation of the Cα by the ether oxygen proceeds with an activation barrier of 77.86 kcalmol^-1^ in the gas phase and 64.3 kcalmol^-1^ in the solavated phase and the products were formed with a reaction energy of -1.85 kcalmol^-1^ and -5.6 kcalmol^-1^ in the gas phase and the solvated phase respectively. These energies show that this pathway requires extreme experimental conditions and thus the pathway for the C-O bind cleavage via the 6-memebered transition state in which both sodium ion and hydroxide ion participates is the kinetically and thermodynamically preferred pathway for the base catalyzed cleavage of lignin the Na ion goes on to protonate the ether oxygen of the β-O-4 substrate forming a cation adduct.





Figure S1. Free energy profile of the reaction between metal ion and C2 *β*-O-4 substrate.

Table S1. Electronic energies (EE) and relative energies (RE) of the 6-31G* and 6-311G* optimized geometries of the parent reaction between the C2 substrate and NaOH.

|  | 6-31G* EE (au) | 6-311G* EE (kcal/mol) | 6-31G* RE (kcal/mol) | 6-311G* RE (kcal/mol) | Diff in RE | Diff in Ea/Ef |
| --- | --- | --- | --- | --- | --- | --- |
| Reactants | -1541.142222 | -1541.44441 |  |  |  |  |
| INT1 | -1541.179048 | -1541.485066 | -23.108315 | -25.51164 | 2.40 | 2.40 |
| TS1 | -1541.1612 | -1541.457249 | -11.908695 | -8.0564725 | 3.85 | 6.26 |
| Products | -1541.188826 | -1541.492814 | -29.24401 | -30.37351 | 1.12 | 1.37 |

Table S2**.** The Cartesian coordinates, total energy, zero-point vibrational energy and number of imaginary frequencies, of the optimized structures in Figure 2.

Keywords for Reactants, Intermediates, Products: #n opt freq m06/6-31g(d) test

Keywords for Transition states: #n M06/6-31G(d) Opt=(calcfc,tight,ts,noeigen) freq int=ultrafine test

| **A1+NaOH**  **A1**  E(M06) = -1303.455988 Hartrees.  ZPVE = 144.0872275 kcal mol^-1^  NImag = 0  H -4.24575 -0.48267 -2.22194  C -3.82513 -0.23280 -1.24827  C -2.72580 0.32308 1.24251  C -2.88354 -1.10916 -0.69997  C -4.21007 0.91531 -0.57299  C -3.66296 1.18736 0.68568  C -2.31008 -0.82697 0.54299  H -3.98113 2.08114 1.22086  O -2.53828 -2.18191 -1.45511  O -2.12763 0.51272 2.44614  C -2.44191 -3.45375 -0.82211  H -1.43884 -3.62196 -0.41901  H -2.66564 -4.19318 -1.59871  H -3.18268 -3.54291 -0.01606  C -2.50269 1.64578 3.18635  H -2.27887 2.57774 2.64473  H -1.91809 1.61819 4.10952  H -3.57401 1.62970 3.43677  C -5.26435 1.82314 -1.14398  H -5.25442 1.75416 -2.24135  H -5.02978 2.86920 -0.89486  C -6.65519 1.47481 -0.62257  H -6.91892 0.44161 -0.88388  H -7.42125 2.14008 -1.04012  H -6.69355 1.55587 0.47193  O -1.37969 -1.66439 1.09778  C -0.04488 -1.18664 1.03781  H 0.53512 -1.79919 1.74092  C 0.57367 -1.32346 -0.34231  H -0.11806 -0.86224 -1.07392  C 1.89611 -0.59716 -0.38052  C 4.33316 0.77884 -0.33308  C 1.92640 0.76838 -0.68227  C 3.06977 -1.25358 -0.04118  C 4.29232 -0.57867 -0.01449  C 3.13643 1.44298 -0.64941  H 1.00045 1.28119 -0.94039  H 3.05828 -2.31262 0.20859  H 0.00279 -0.13766 1.36613  O 0.70604 -2.70678 -0.59218  H 1.08737 -2.81011 -1.47522  O 5.40205 -1.30924 0.27406  O 3.31950 2.76734 -0.93537  O 5.50930 1.45726 -0.36372  H 5.29502 2.37153 -0.61315  C 6.31552 -0.77506 1.21500  H 6.95992 -0.00828 0.77181  H 6.92536 -1.61637 1.55822  H 5.78464 -0.34116 2.07511  C 2.18926 3.51569 -1.31120  H 2.53767 4.53468 -1.49722  H 1.43582 3.53008 -0.51016  H 1.73181 3.11448 -2.22735  **NaOH**  E(M06) = -238.055709 Hartrees.  ZPVE = 6.7224075 kcal mol^-1^  NImag = 0  O 0.02821 1.03311 0.00000  H -0.53591 1.80813 0.00000  Na 0.02821 -0.91573 0.00000 | **A2^NaOH^**  E(M06) = -1541.571517 Hartrees.  ZPVE = 284.622705 kcal mol^-1^  NImag = 0  H -4.95735 0.20137 -0.75746  C -3.96515 0.27616 -0.31593  C -1.44642 0.49588 0.84794  C -3.10478 -0.82092 -0.31732  C -3.56966 1.48268 0.25934  C -2.31492 1.58604 0.85245  C -1.82256 -0.70599 0.23972  H -2.01921 2.52566 1.31556  C -4.52565 2.64206 0.31328  H -3.96148 3.58577 0.29910  H -5.15769 2.64416 -0.58670  C -5.40617 2.58874 1.55819  H -4.79390 2.60525 2.46957  H -5.99857 1.66435 1.57781  H -6.09888 3.43820 1.59742  O -0.95149 -1.76526 0.24529  C -0.55984 -2.27249 -1.04052  H -0.90730 -1.59063 -1.82820  H -1.02914 -3.25060 -1.19775  C 0.95261 -2.39928 -1.12203  C 1.66279 -1.08322 -0.93203  C 2.90471 1.37804 -0.50198  C 1.42425 -0.01510 -1.79730  C 2.56766 -0.94573 0.12445  C 3.17155 0.28785 0.33597  C 2.03964 1.21539 -1.58999  H 2.76388 -1.79521 0.80190  O 1.15788 -2.95417 -2.41054  H 2.10965 -2.93610 -2.57906  Na 0.61240 -1.67905 2.04378  O -3.40420 -2.03203 -0.84167  O -0.22015 0.50947 1.44909  C 0.37151 1.77415 1.69444  H 0.34660 2.39610 0.78950  H 1.41641 1.58162 1.95747  H -0.12595 2.29462 2.52466  C -4.66644 -2.19179 -1.44504  H -4.70121 -3.21971 -1.81322  H -4.80058 -1.49886 -2.28839  H -5.47917 -2.03900 -0.72007  H 1.29918 -3.09320 -0.33113  O 3.44532 2.59043 -0.21770  O 1.71817 2.25934 -2.40945  O 2.32185 -2.77023 2.39765  H 2.63025 -3.53682 2.88823  H 0.75084 -0.11148 -2.64854  C 2.81201 2.85030 -3.08849  H 2.38947 3.59119 -3.77356  H 3.36061 2.09426 -3.67056  H 3.50399 3.34219 -2.39374  H 4.01260 2.44002 0.56074  O 4.05361 0.57599 1.34171  C 3.85961 -0.06671 2.60178  H 3.03153 0.42596 3.14207  H 4.78429 0.08812 3.16529  H 3.61817 -1.14258 2.52090 | **TS1^NaOH^**  E(M06) = -1541.542243 Hartrees.  ZPVE = 279.750795 kcal mol^-1^  NImag = 1  H -5.22111 -0.79329 -1.31512  C -4.37943 -0.36401 -0.77432  C -2.26227 0.75905 0.62180  C -3.26059 -1.14065 -0.47579  C -4.44805 0.96928 -0.37182  C -3.38931 1.52667 0.33870  C -2.17158 -0.57435 0.20817  H -3.45157 2.56235 0.66953  C -5.69213 1.77293 -0.63319  H -5.43052 2.83522 -0.74657  H -6.14199 1.46058 -1.58705  C -6.71265 1.61422 0.49013  H -6.29041 1.94380 1.44872  H -7.00454 0.56196 0.60555  H -7.61962 2.20040 0.29731  O -1.06215 -1.28916 0.53891  C -0.27884 -1.88488 -0.56315  H -0.42348 -1.21276 -1.42982  H -0.70492 -2.86393 -0.80156  C 1.13640 -2.05302 -0.10205  C 2.02572 -0.89442 -0.30729  C 3.78510 1.33679 -0.34616  C 1.54537 0.42408 -0.38518  C 3.42288 -1.07261 -0.28513  C 4.27192 0.01849 -0.31501  C 2.40998 1.52407 -0.39709  H 3.80487 -2.08916 -0.25901  O 1.70261 -3.27108 -0.59381  H 1.89058 -3.81302 0.18449  Na 0.46613 -0.31039 2.07817  O -3.12893 -2.44779 -0.80107  O -1.19129 1.24423 1.33267  C -0.95969 2.64351 1.27597  H -1.06454 3.00943 0.24658  H 0.07486 2.80871 1.59162  H -1.64426 3.18816 1.94035  C -4.16986 -3.05464 -1.52667  H -3.86102 -4.08884 -1.69673  H -4.32805 -2.56031 -2.49666  H -5.11371 -3.04806 -0.96144  H 1.14016 -2.24103 1.28792  O 4.64617 2.39467 -0.31512  O 1.85495 2.77967 -0.40966  O 1.26075 -2.30088 2.50505  H 0.58672 -2.93533 2.78350  H 0.47913 0.63971 -0.47165  C 2.20357 3.57076 -1.53127  H 1.61602 4.49186 -1.46063  H 1.94694 3.05028 -2.46670  H 3.27230 3.81748 -1.53605  H 5.53877 2.01330 -0.28182  O 5.63853 -0.04123 -0.29229  C 6.23172 -1.31297 -0.19166  H 5.91749 -1.82738 0.72818  H 7.31270 -1.15122 -0.16726  H 5.97710 -1.94176 -1.05693 |
| --- | --- | --- |
| **A3^NaOH^**  **(Phenolate)**  E(M06) = -776.456172 Hartrees.  ZPVE = 134.50086 kcal mol^-1^  NImag = 0  H 2.23307 1.82621 -0.14401  C 1.49784 1.01878 -0.13890  C -0.40780 -0.93954 -0.10504  C 0.17013 1.36980 0.04202  C 1.90932 -0.31073 -0.29444  C 0.92389 -1.29157 -0.28012  C -0.87016 0.39180 0.07701  H 1.17678 -2.34715 -0.40244  O -0.11785 2.69381 0.25184  O -1.37575 -1.96766 -0.07369  C -1.03419 3.24996 -0.66955  H -0.64372 3.17477 -1.69793  H -1.13933 4.30846 -0.40813  H -2.00756 2.74791 -0.61194  C -1.94630 -2.16540 -1.35853  H -2.69997 -2.95825 -1.27308  H -1.17396 -2.48120 -2.07378  H -2.41414 -1.23740 -1.71876  C 3.36687 -0.65741 -0.41772  H 3.47524 -1.63408 -0.91394  H 3.87240 0.07429 -1.06709  C 4.06930 -0.69513 0.93638  H 5.13620 -0.93629 0.83919  H 3.60662 -1.44620 1.59099  H 3.98390 0.27594 1.44256  O -2.11795 0.68075 0.29268  Na -2.78059 -0.94959 1.44773  **(Hydroxylvinyl)**  E(M06) = -688.699461 Hartrees.  ZPVE = 130.55765 kcal mol^-1^  NImag = 0  C -3.64749 0.24098 0.73692  H -4.70904 0.01179 0.72279  C -2.78272 -0.51950 0.05804  C -1.33123 -0.27564 -0.01673  C 1.43669 0.16770 -0.15835  C -0.82810 1.03261 0.01100  C -0.44071 -1.34387 -0.11571  C 0.93511 -1.13293 -0.18530  C 0.53882 1.24297 -0.04910  H -1.52494 1.86604 0.04841  H -0.78808 -2.37562 -0.09955  H -3.30189 1.06270 1.35580  O -3.23980 -1.63418 -0.58558  H -2.60603 -1.87192 -1.27771  O 1.75300 -2.21381 -0.32125  O 1.15929 2.45885 -0.04925  O 2.76698 0.39772 -0.25642  H 2.88816 1.36156 -0.22825  C 2.66801 -2.41915 0.74056  H 3.41445 -1.61754 0.79622  H 3.16903 -3.36995 0.53737  H 2.13705 -2.49011 1.70191  C 0.34603 3.60715 -0.00232  H 1.01976 4.46727 -0.01224  H -0.25893 3.62898 0.91571  H -0.32190 3.65630 -0.87452 | **TS2^NaOH^**  E(M06) = -688.623968 Hartrees.  ZPVE = 126881755 kcal mol^-1^  NImag = 1  C -3.81814 0.19643 0.34299  H -4.46995 0.37193 -0.51953  C -2.70182 -0.62330 0.00741  C -1.28261 -0.33472 -0.04414  C 1.44732 0.20686 -0.17227  C -0.83659 0.99561 0.02050  C -0.36209 -1.37894 -0.15801  C 0.99896 -1.11745 -0.23042  C 0.51859 1.25554 -0.03512  H -1.56870 1.79610 0.09502  H -0.70603 -2.40976 -0.19391  H -3.70639 1.08031 0.97478  O -3.14393 -1.82021 -0.21625  H -4.13854 -1.25909 0.29052  O 1.87134 -2.14840 -0.39767  O 1.10602 2.48607 0.00703  O 2.76302 0.48052 -0.25270  H 2.85658 1.44771 -0.20266  C 2.75296 -2.37310 0.68897  H 3.44754 -1.53512 0.82890  H 3.31901 -3.27656 0.44607  H 2.18853 -2.53970 1.61881  C 0.25895 3.60763 0.10470  H 0.90726 4.48679 0.12186  H -0.33635 3.57587 1.02863  H -0.41774 3.67020 -0.75968 | **A4I^NaOH^**  E(M06) = -614.735882 Hartrees.  ZPVE = 142.066 kcal mol^-1^  NImag = 0  H -1.98374 1.50263 -0.34465  C -1.11205 0.85523 -0.25965  C 1.14136 -0.79131 -0.03822  C -1.25316 -0.51235 -0.04498  C 0.16390 1.40660 -0.36554  C 1.28162 0.58061 -0.25336  C -0.13772 -1.34615 0.06701  H 2.27276 1.02342 -0.33720  C 0.33317 2.89242 -0.52645  H 1.25816 3.10296 -1.08357  H -0.49125 3.29737 -1.13236  C 0.37442 3.60827 0.82047  H 1.20720 3.23650 1.43217  H -0.55065 3.42764 1.38416  H 0.49534 4.69243 0.70120  O -0.27817 -2.67842 0.26897  O -2.44065 -1.18151 0.07184  O 2.16827 -1.66781 0.07807  C 3.46908 -1.15187 -0.02434  H 3.64490 -0.68467 -1.00579  H 4.14815 -1.99997 0.09553  H 3.67543 -0.41005 0.76277  C -3.62585 -0.43446 -0.03353  H -4.45066 -1.14116 0.08948  H -3.70913 0.05058 -1.01753  H -3.68810 0.33408 0.75121  H -1.23263 -2.85181 0.30505  **A4II^NaOH^**  E(M06) = -688.731116 Hartrees.  ZPVE = 130.330495 kcal mol^-1^  NImag = 0  C -2.66249 -0.94940 0.00939  C -1.25704 -0.47070 -0.04904  C 1.42343 0.31316 -0.16909  C -0.92500 0.88965 0.03500  C -0.24552 -1.42015 -0.18405  C 1.08791 -1.04219 -0.24698  C 0.40491 1.26993 -0.02298  H -1.70301 1.64097 0.14641  H -0.50632 -2.47395 -0.25097  O -2.92685 -2.13636 -0.05262  O 2.04877 -1.98964 -0.43534  O 0.87865 2.55024 0.03969  O 2.71342 0.69984 -0.24301  H 2.72283 1.67025 -0.18428  C 2.90077 -2.19976 0.67587  H 3.50658 -1.31179 0.89964  H 3.56117 -3.02951 0.40881  H 2.31693 -2.47620 1.56717  C -0.06200 3.59281 0.12509  H 0.50735 4.52540 0.13866  H -0.65789 3.52006 1.04674  H -0.73719 3.59240 -0.74318  C -3.76373 0.07527 0.15028  H -3.75051 0.79179 -0.68156  H -4.72549 -0.44398 0.16242  H -3.65382 0.65021 1.07944 |

Table S3**.** The Cartesian coordinates, total energy, zero-point vibrational energy and number of imaginary frequencies, of the optimized structures in Figure 3.

| **A1+KOH**  **A1**  E(M06) = -1303.455988 Hartrees.  ZPVE = 144.0872275 kcal mol^-1^  NImag = 0  H -4.24575 -0.48267 -2.22194  C -3.82513 -0.23280 -1.24827  C -2.72580 0.32308 1.24251  C -2.88354 -1.10916 -0.69997  C -4.21007 0.91531 -0.57299  C -3.66296 1.18736 0.68568  C -2.31008 -0.82697 0.54299  H -3.98113 2.08114 1.22086  O -2.53828 -2.18191 -1.45511  O -2.12763 0.51272 2.44614  C -2.44191 -3.45375 -0.82211  H -1.43884 -3.62196 -0.41901  H -2.66564 -4.19318 -1.59871  H -3.18268 -3.54291 -0.01606  C -2.50269 1.64578 3.18635  H -2.27887 2.57774 2.64473  H -1.91809 1.61819 4.10952  H -3.57401 1.62970 3.43677  C -5.26435 1.82314 -1.14398  H -5.25442 1.75416 -2.24135  H -5.02978 2.86920 -0.89486  C -6.65519 1.47481 -0.62257  H -6.91892 0.44161 -0.88388  H -7.42125 2.14008 -1.04012  H -6.69355 1.55587 0.47193  O -1.37969 -1.66439 1.09778  C -0.04488 -1.18664 1.03781  H 0.53512 -1.79919 1.74092  C 0.57367 -1.32346 -0.34231  H -0.11806 -0.86224 -1.07392  C 1.89611 -0.59716 -0.38052  C 4.33316 0.77884 -0.33308  C 1.92640 0.76838 -0.68227  C 3.06977 -1.25358 -0.04118  C 4.29232 -0.57867 -0.01449  C 3.13643 1.44298 -0.64941  H 1.00045 1.28119 -0.94039  H 3.05828 -2.31262 0.20859  H 0.00279 -0.13766 1.36613  O 0.70604 -2.70678 -0.59218  H 1.08737 -2.81011 -1.47522  O 5.40205 -1.30924 0.27406  O 3.31950 2.76734 -0.93537  O 5.50930 1.45726 -0.36372  H 5.29502 2.37153 -0.61315  C 6.31552 -0.77506 1.21500  H 6.95992 -0.00828 0.77181  H 6.92536 -1.61637 1.55822  H 5.78464 -0.34116 2.07511  C 2.18926 3.51569 -1.31120  H 2.53767 4.53468 -1.49722  H 1.43582 3.53008 -0.51016  H 1.73181 3.11448 -2.22735  **KOH**  E(M06) = -675.655106 Hartrees.  ZPVE = 6.7111125 kcal mol^-1^  NImag = 0  O 0.00005 1.46874 0.00000  H -0.00130 2.42776 0.00000  K 0.00005 -0.74619 0.00000 | **A2^KOH^**  E(M06) = -1979.166015 Hartrees.  ZPVE = 285.039365 kcal mol^-1^  NImag = 0  H 4.99029 0.01484 0.56940  C 3.97737 0.11682 0.18405  C 1.39312 0.40197 -0.83144  C 3.05253 -0.91434 0.33774  C 3.61471 1.29143 -0.47182  C 2.33013 1.42463 -0.98963  C 1.74107 -0.76416 -0.13804  H 2.06208 2.34360 -1.50672  C 4.63280 2.37774 -0.68194  H 4.12654 3.35134 -0.75114  H 5.29817 2.43428 0.19200  C 5.46152 2.13922 -1.94083  H 4.81786 2.10714 -2.82997  H 5.98964 1.17810 -1.88318  H 6.20819 2.92909 -2.08875  O 0.81811 -1.76400 0.02660  C 0.49760 -2.10581 1.38275  H 0.98340 -1.40118 2.07029  H 0.88428 -3.10925 1.59806  C -1.00463 -2.05279 1.61278  C -1.60165 -0.72119 1.23923  C -2.62746 1.73561 0.40870  C -1.12009 0.46373 1.79679  C -2.62907 -0.68978 0.29429  C -3.12661 0.53934 -0.11812  C -1.61941 1.69092 1.37736  H -2.97914 -1.63734 -0.16955  O -1.12661 -2.34470 2.99682  H -2.05765 -2.22654 3.22859  O 3.31734 -2.09767 0.94297  O 0.13228 0.45345 -1.34391  C -0.32253 1.70450 -1.82313  H -0.24157 2.47879 -1.04707  H -1.38006 1.57472 -2.07331  H 0.23103 2.01419 -2.72205  C 4.59799 -2.27753 1.49809  H 4.60538 -3.27998 1.93261  H 4.80283 -1.53862 2.28674  H 5.38304 -2.21016 0.73052  H -1.50495 -2.83048 1.00243  O -3.07650 2.92700 -0.06397  O -1.05082 2.84527 1.84458  O -3.00591 -3.06474 -1.41337  H -3.77458 -3.60088 -1.19785  H -0.32779 0.46122 2.54548  C -1.90475 3.63745 2.64801  H -1.31922 4.50419 2.96942  H -2.23448 3.07574 3.53564  H -2.78509 3.98195 2.08958  H -3.77325 2.69996 -0.70613  O -4.11768 0.71525 -1.05022  C -4.13647 -0.18180 -2.15744  H -3.35536 0.11288 -2.88307  H -5.11234 -0.05665 -2.63639  H -3.97565 -1.24125 -1.87304  K -0.94317 -2.12549 -1.98226 | **TS1^KOH^**  E(M06) = -1979.140724 Hartrees.  ZPVE = 280.0601525 kcal mol^-1^  NImag = 1  H -5.32924 -0.78051 -1.26701  C -4.46752 -0.34726 -0.76201  C -2.29641 0.78535 0.54543  C -3.33018 -1.11579 -0.51665  C -4.52651 0.98433 -0.35277  C -3.44097 1.54558 0.31255  C -2.21458 -0.54590 0.12063  H -3.49472 2.58071 0.64719  C -5.78552 1.78110 -0.55783  H -5.53579 2.84592 -0.67480  H -6.27216 1.47171 -1.49455  C -6.75883 1.60882 0.60469  H -6.30010 1.93570 1.54738  H -7.03847 0.55377 0.72528  H -7.67707 2.18963 0.45271  O -1.08569 -1.25483 0.38698  C -0.35210 -1.80691 -0.76307  H -0.52236 -1.10517 -1.60175  H -0.78494 -2.77740 -1.02533  C 1.07468 -1.99350 -0.35484  C 1.96407 -0.82734 -0.50487  C 3.73494 1.39113 -0.39797  C 1.49206 0.49407 -0.56472  C 3.35900 -1.01445 -0.42025  C 4.21594 0.07114 -0.37778  C 2.36244 1.58664 -0.50458  H 3.73271 -2.03447 -0.39785  O 1.63868 -3.18882 -0.89831  H 1.87654 -3.71878 -0.12058  O -3.20406 -2.42084 -0.85556  O -1.20238 1.27293 1.21134  C -0.98367 2.67036 1.15852  H -1.11810 3.04786 0.13606  H 0.05941 2.84031 1.44541  H -1.65101 3.21050 1.84540  C -4.27011 -3.02949 -1.54084  H -3.96138 -4.05992 -1.73310  H -4.47404 -2.52844 -2.49893  H -5.18938 -3.03530 -0.93613  H 1.12085 -2.36438 0.94834  O 4.59514 2.44634 -0.30997  O 1.82585 2.85128 -0.50801  O 1.43142 -2.81446 2.07534  H 0.78521 -3.51260 2.24365  H 0.42758 0.71506 -0.64839  C 2.17443 3.62684 -1.64024  H 1.62178 4.56850 -1.56033  H 1.87879 3.11198 -2.56733  H 3.25107 3.83706 -1.66913  H 5.48702 2.06346 -0.27466  O 5.57986 0.00218 -0.29025  C 6.16229 -1.27795 -0.23859  H 5.81412 -1.83945 0.64066  H 7.24262 -1.12572 -0.16846  H 5.93323 -1.85709 -1.14462  K 0.77931 -0.36336 2.27312 |
| --- | --- | --- |
| **A3^KOH^**  **(Phenolate)**  E(M06) = -1214.059934 Hartrees.  ZPVE = 134.5755325 kcal mol^-1^  NImag = 0  H -2.55487 1.66800 -0.14470  C -1.76174 0.93885 0.03529  C 0.29096 -0.82181 0.46910  C -0.45471 1.39278 -0.02614  C -2.07866 -0.39959 0.30065  C -1.02150 -1.27534 0.52582  C 0.66321 0.52304 0.17858  H -1.20049 -2.32947 0.74920  O -0.24792 2.70589 -0.37050  O 1.32546 -1.75438 0.65300  C 0.48835 3.44858 0.58048  H -0.04905 3.48871 1.54240  H 0.58053 4.46576 0.18432  H 1.48268 3.01255 0.73464  C 1.94392 -1.59535 1.91977  H 2.73115 -2.35497 2.00268  H 1.21055 -1.75179 2.72416  H 2.37856 -0.58992 2.00964  C -3.50706 -0.86857 0.29387  H -3.59220 -1.80376 0.86830  H -4.14617 -0.13367 0.80825  C -4.03999 -1.09129 -1.11853  H -5.08720 -1.42226 -1.11597  H -3.44269 -1.85120 -1.64099  H -3.97787 -0.16528 -1.70612  O 1.89684 0.88995 0.05775  K 2.56502 -0.86696 -1.51006  **(Hydroxylvinyl)**  E(M06) = -688.699461 Hartrees.  ZPVE = 130.55765 kcal mol^-1^  NImag = 0  C -3.64749 0.24098 0.73692  H -4.70904 0.01179 0.72279  C -2.78272 -0.51950 0.05804  C -1.33123 -0.27564 -0.01673  C 1.43669 0.16770 -0.15835  C -0.82810 1.03261 0.01100  C -0.44071 -1.34387 -0.11571  C 0.93511 -1.13293 -0.18530  C 0.53882 1.24297 -0.04910  H -1.52494 1.86604 0.04841  H -0.78808 -2.37562 -0.09955  H -3.30189 1.06270 1.35580  O -3.23980 -1.63418 -0.58558  H -2.60603 -1.87192 -1.27771  O 1.75300 -2.21381 -0.32125  O 1.15929 2.45885 -0.04925  O 2.76698 0.39772 -0.25642  H 2.88816 1.36156 -0.22825  C 2.66801 -2.41915 0.74056  H 3.41445 -1.61754 0.79622  H 3.16903 -3.36995 0.53737  H 2.13705 -2.49011 1.70191  C 0.34603 3.60715 -0.00232  H 1.01976 4.46727 -0.01224  H -0.25893 3.62898 0.91571  H -0.32190 3.65630 -0.87452 | **TS2^KOH^**  E(M06) = -688.623968 Hartrees.  ZPVE = 126881755 kcal mol^-1^  NImag = 1  C -3.81814 0.19643 0.34299  H -4.46995 0.37193 -0.51953  C -2.70182 -0.62330 0.00741  C -1.28261 -0.33472 -0.04414  C 1.44732 0.20686 -0.17227  C -0.83659 0.99561 0.02050  C -0.36209 -1.37894 -0.15801  C 0.99896 -1.11745 -0.23042  C 0.51859 1.25554 -0.03512  H -1.56870 1.79610 0.09502  H -0.70603 -2.40976 -0.19391  H -3.70639 1.08031 0.97478  O -3.14393 -1.82021 -0.21625  H -4.13854 -1.25909 0.29052  O 1.87134 -2.14840 -0.39767  O 1.10602 2.48607 0.00703  O 2.76302 0.48052 -0.25270  H 2.85658 1.44771 -0.20266  C 2.75296 -2.37310 0.68897  H 3.44754 -1.53512 0.82890  H 3.31901 -3.27656 0.44607  H 2.18853 -2.53970 1.61881  C 0.25895 3.60763 0.10470  H 0.90726 4.48679 0.12186  H -0.33635 3.57587 1.02863  H -0.41774 3.67020 -0.75968 | **A4I^KOH^**  E(M06) = -614.735882 Hartrees.  ZPVE = 142.066 kcal mol^-1^  NImag = 0  H -1.98374 1.50263 -0.34465  C -1.11205 0.85523 -0.25965  C 1.14136 -0.79131 -0.03822  C -1.25316 -0.51235 -0.04498  C 0.16390 1.40660 -0.36554  C 1.28162 0.58061 -0.25336  C -0.13772 -1.34615 0.06701  H 2.27276 1.02342 -0.33720  C 0.33317 2.89242 -0.52645  H 1.25816 3.10296 -1.08357  H -0.49125 3.29737 -1.13236  C 0.37442 3.60827 0.82047  H 1.20720 3.23650 1.43217  H -0.55065 3.42764 1.38416  H 0.49534 4.69243 0.70120  O -0.27817 -2.67842 0.26897  O -2.44065 -1.18151 0.07184  O 2.16827 -1.66781 0.07807  C 3.46908 -1.15187 -0.02434  H 3.64490 -0.68467 -1.00579  H 4.14815 -1.99997 0.09553  H 3.67543 -0.41005 0.76277  C -3.62585 -0.43446 -0.03353  H -4.45066 -1.14116 0.08948  H -3.70913 0.05058 -1.01753  H -3.68810 0.33408 0.75121  H -1.23263 -2.85181 0.30505  **A4II^KOH^**  E(M06) = -688.731116 Hartrees.  ZPVE = 130.330495 kcal mol^-1^  NImag = 0  C -2.66249 -0.94940 0.00939  C -1.25704 -0.47070 -0.04904  C 1.42343 0.31316 -0.16909  C -0.92500 0.88965 0.03500  C -0.24552 -1.42015 -0.18405  C 1.08791 -1.04219 -0.24698  C 0.40491 1.26993 -0.02298  H -1.70301 1.64097 0.14641  H -0.50632 -2.47395 -0.25097  O -2.92685 -2.13636 -0.05262  O 2.04877 -1.98964 -0.43534  O 0.87865 2.55024 0.03969  O 2.71342 0.69984 -0.24301  H 2.72283 1.67025 -0.18428  C 2.90077 -2.19976 0.67587  H 3.50658 -1.31179 0.89964  H 3.56117 -3.02951 0.40881  H 2.31693 -2.47620 1.56717  C -0.06200 3.59281 0.12509  H 0.50735 4.52540 0.13866  H -0.65789 3.52006 1.04674  H -0.73719 3.59240 -0.74318  C -3.76373 0.07527 0.15028  H -3.75051 0.79179 -0.68156  H -4.72549 -0.44398 0.16242  H -3.65382 0.65021 1.07944 |

Table S4**.** The Cartesian coordinates, total energy, zero-point vibrational energy and number of imaginary frequencies, of the optimized structures in Figure 4.

| **A1+LiOH**  **A1**  E(M06) = -1303.455988 Hartrees.  ZPVE = 144.0872275 kcal mol^-1^  NImag = 0  H -4.24575 -0.48267 -2.22194  C -3.82513 -0.23280 -1.24827  C -2.72580 0.32308 1.24251  C -2.88354 -1.10916 -0.69997  C -4.21007 0.91531 -0.57299  C -3.66296 1.18736 0.68568  C -2.31008 -0.82697 0.54299  H -3.98113 2.08114 1.22086  O -2.53828 -2.18191 -1.45511  O -2.12763 0.51272 2.44614  C -2.44191 -3.45375 -0.82211  H -1.43884 -3.62196 -0.41901  H -2.66564 -4.19318 -1.59871  H -3.18268 -3.54291 -0.01606  C -2.50269 1.64578 3.18635  H -2.27887 2.57774 2.64473  H -1.91809 1.61819 4.10952  H -3.57401 1.62970 3.43677  C -5.26435 1.82314 -1.14398  H -5.25442 1.75416 -2.24135  H -5.02978 2.86920 -0.89486  C -6.65519 1.47481 -0.62257  H -6.91892 0.44161 -0.88388  H -7.42125 2.14008 -1.04012  H -6.69355 1.55587 0.47193  O -1.37969 -1.66439 1.09778  C -0.04488 -1.18664 1.03781  H 0.53512 -1.79919 1.74092  C 0.57367 -1.32346 -0.34231  H -0.11806 -0.86224 -1.07392  C 1.89611 -0.59716 -0.38052  C 4.33316 0.77884 -0.33308  C 1.92640 0.76838 -0.68227  C 3.06977 -1.25358 -0.04118  C 4.29232 -0.57867 -0.01449  C 3.13643 1.44298 -0.64941  H 1.00045 1.28119 -0.94039  H 3.05828 -2.31262 0.20859  H 0.00279 -0.13766 1.36613  O 0.70604 -2.70678 -0.59218  H 1.08737 -2.81011 -1.47522  O 5.40205 -1.30924 0.27406  O 3.31950 2.76734 -0.93537  O 5.50930 1.45726 -0.36372  H 5.29502 2.37153 -0.61315  C 6.31552 -0.77506 1.21500  H 6.95992 -0.00828 0.77181  H 6.92536 -1.61637 1.55822  H 5.78464 -0.34116 2.07511  C 2.18926 3.51569 -1.31120  H 2.53767 4.53468 -1.49722  H 1.43582 3.53008 -0.51016  H 1.73181 3.11448 -2.22735  **LiOH**  E(M06) = -83.331306 Hartrees.  ZPVE = 7.3009625 kcal mol^-1^  NImag = 0  O 0.00014 -0.31789 0.00000  H -0.00154 -1.27188 0.00000  Li 0.00014 1.27166 0.00000 | **A2^LiOH^**  E(M06) = -1386.854515 Hartrees.  ZPVE = 285.5834075 kcal mol^-1^  NImag = 0  H -4.90354 0.03545 0.59783  C -3.89155 -0.12991 0.23331  C -1.32348 -0.58852 -0.72605  C -3.01762 0.94451 0.06034  C -3.48435 -1.42774 -0.06798  C -2.20067 -1.65549 -0.56074  C -1.71238 0.71123 -0.38782  H -1.89312 -2.66963 -0.80824  C -4.45086 -2.57194 0.06384  H -3.90957 -3.47993 0.36726  H -5.17214 -2.35722 0.86560  C -5.19706 -2.83191 -1.24164  H -4.49450 -3.06940 -2.05131  H -5.76382 -1.94332 -1.54990  H -5.90053 -3.66782 -1.14420  O -0.81107 1.73181 -0.57428  C -0.50673 2.52789 0.57970  H -0.90065 2.04025 1.48088  H -0.98835 3.50719 0.48063  C 0.99967 2.68430 0.72802  C 1.70710 1.35306 0.72511  C 2.83273 -1.19724 0.55760  C 1.36163 0.37003 1.64986  C 2.65517 1.07767 -0.26568  C 3.20377 -0.19821 -0.35447  C 1.91291 -0.90545 1.56689  H 2.88308 1.82313 -1.02797  O 1.13837 3.39019 1.94692  H 2.08101 3.41798 2.15913  O -3.33106 2.23900 0.30422  O -0.06847 -0.69940 -1.24193  C 0.45592 -1.99083 -1.49397  H 0.49629 -2.57937 -0.56662  H 1.47066 -1.83357 -1.87186  H -0.14157 -2.51477 -2.25215  C -4.63067 2.52408 0.76404  H -4.67991 3.60827 0.88888  H -4.83250 2.03781 1.72984  H -5.39186 2.20853 0.03583  H 1.37924 3.28250 -0.12317  O 3.32294 -2.45473 0.41032  O 1.46329 -1.86989 2.42083  O 1.79424 1.55995 -3.10328  H 1.55001 1.80193 -4.00075  H 0.63499 0.56136 2.43840  C 2.44870 -2.46431 3.24711  H 1.91304 -3.09800 3.95999  H 3.00751 -1.69430 3.80006  H 3.15076 -3.07606 2.66769  H 3.94851 -2.41247 -0.33439  O 4.06217 -0.63540 -1.30767  C 4.48942 0.30375 -2.29923  H 5.10928 -0.26940 -2.99370  H 5.10493 1.08354 -1.82602  H 3.61726 0.75533 -2.81093  Li 0.71741 1.06666 -1.87372 | **TS1^LiOH^**  E(M06) = -1386.808682Hartrees.  ZPVE = 2832.0330125 kcal mol^-1^  NImag = 1  H -5.11637 -0.67129 -1.41911  C -4.28122 -0.30991 -0.82003  C -2.18776 0.63763 0.72208  C -3.21399 -1.15547 -0.51616  C -4.30783 1.00408 -0.35403  C -3.25822 1.47593 0.43099  C -2.13582 -0.67468 0.24182  H -3.28543 2.49679 0.80675  C -5.49808 1.87872 -0.63244  H -5.18060 2.93022 -0.68753  H -5.92086 1.63024 -1.61651  C -6.57375 1.72523 0.44046  H -6.17798 1.99358 1.42894  H -6.92101 0.68511 0.49669  H -7.44052 2.36370 0.23598  O -1.05790 -1.44034 0.57541  C -0.26001 -2.05653 -0.55048  H -0.47915 -1.39592 -1.41293  H -0.67743 -3.04773 -0.74597  C 1.15640 -2.16120 -0.13484  C 1.98649 -0.98891 -0.32078  C 3.62677 1.34938 -0.39800  C 1.43983 0.31193 -0.42715  C 3.39996 -1.08162 -0.29149  C 4.18097 0.05503 -0.34041  C 2.24641 1.45611 -0.46673  H 3.84282 -2.07350 -0.25750  O 1.76620 -3.38585 -0.54463  H 1.88581 -3.93422 0.23766  O -3.12594 -2.44863 -0.90565  O -1.12526 1.01662 1.51279  C -0.83264 2.40664 1.59606  H -0.76625 2.84275 0.59196  H 0.14601 2.48989 2.07501  H -1.58324 2.92897 2.20250  C -4.15673 -2.96620 -1.70973  H -3.88541 -4.00275 -1.92512  H -4.24742 -2.41206 -2.65641  H -5.12402 -2.94688 -1.18895  H 1.19250 -2.12603 1.70106  O 4.43427 2.45211 -0.38085  O 1.61475 2.67376 -0.53533  O 1.11566 -1.76273 2.65070  H 0.42837 -2.30811 3.06108  H 0.37393 0.47203 -0.60766  C 1.96946 3.46469 -1.65443  H 1.31780 4.34458 -1.63626  H 1.79949 2.90908 -2.58931  H 3.01736 3.78322 -1.61038  H 5.34411 2.11648 -0.33645  O 5.54887 0.07167 -0.31568  C 6.21310 -1.16244 -0.20571  H 5.93166 -1.68463 0.71981  H 7.28344 -0.93992 -0.18587  H 5.99289 -1.81376 -1.06382  Li 0.30814 -0.37823 1.58293 |
| --- | --- | --- |
| **A3^LiOH^**  **(Phenolate)**  E(M06) = -621.726522 Hartrees.  ZPVE = 135.965445 kcal mol^-1^  NImag = 0  H 0.72226 2.60521 -0.12507  C 0.49910 1.53733 -0.11128  C -0.14668 -1.11992 -0.05524  C -0.81488 1.16077 0.14365  C 1.50596 0.60214 -0.33542  C 1.16480 -0.75279 -0.30760  C -1.19831 -0.20102 0.19316  H 1.94052 -1.49797 -0.48424  O -1.72494 2.15170 0.39896  O -0.57329 -2.46383 0.00493  C -2.81723 2.19164 -0.49529  H -2.46781 2.34482 -1.52981  H -3.43435 3.04688 -0.20010  H -3.40901 1.26993 -0.44509  C 0.40539 -3.45855 -0.19106  H -0.09332 -4.42806 -0.09408  H 1.20003 -3.38202 0.56373  H 0.85233 -3.38009 -1.19169  C 2.93335 1.02562 -0.54498  H 3.42831 0.33987 -1.25054  H 2.95861 2.01983 -1.01571  C 3.72174 1.06343 0.76075  H 4.76294 1.37368 0.60179  H 3.72972 0.07374 1.23771  H 3.26001 1.76312 1.47002  O -2.41139 -0.61157 0.45123  Li -2.43092 -2.35038 0.42734  **(Hydroxylvinyl)**  E(M06) = -688.699461 Hartrees.  ZPVE = 130.55765 kcal mol^-1^  NImag = 0  C -3.64749 0.24098 0.73692  H -4.70904 0.01179 0.72279  C -2.78272 -0.51950 0.05804  C -1.33123 -0.27564 -0.01673  C 1.43669 0.16770 -0.15835  C -0.82810 1.03261 0.01100  C -0.44071 -1.34387 -0.11571  C 0.93511 -1.13293 -0.18530  C 0.53882 1.24297 -0.04910  H -1.52494 1.86604 0.04841  H -0.78808 -2.37562 -0.09955  H -3.30189 1.06270 1.35580  O -3.23980 -1.63418 -0.58558  H -2.60603 -1.87192 -1.27771  O 1.75300 -2.21381 -0.32125  O 1.15929 2.45885 -0.04925  O 2.76698 0.39772 -0.25642  H 2.88816 1.36156 -0.22825  C 2.66801 -2.41915 0.74056  H 3.41445 -1.61754 0.79622  H 3.16903 -3.36995 0.53737  H 2.13705 -2.49011 1.70191  C 0.34603 3.60715 -0.00232  H 1.01976 4.46727 -0.01224  H -0.25893 3.62898 0.91571  H -0.32190 3.65630 -0.87452 | **TS2^LiOH^**  E(M06) = -688.623968 Hartrees.  ZPVE = 126881755 kcal mol^-1^  NImag = 1  C -3.81814 0.19643 0.34299  H -4.46995 0.37193 -0.51953  C -2.70182 -0.62330 0.00741  C -1.28261 -0.33472 -0.04414  C 1.44732 0.20686 -0.17227  C -0.83659 0.99561 0.02050  C -0.36209 -1.37894 -0.15801  C 0.99896 -1.11745 -0.23042  C 0.51859 1.25554 -0.03512  H -1.56870 1.79610 0.09502  H -0.70603 -2.40976 -0.19391  H -3.70639 1.08031 0.97478  O -3.14393 -1.82021 -0.21625  H -4.13854 -1.25909 0.29052  O 1.87134 -2.14840 -0.39767  O 1.10602 2.48607 0.00703  O 2.76302 0.48052 -0.25270  H 2.85658 1.44771 -0.20266  C 2.75296 -2.37310 0.68897  H 3.44754 -1.53512 0.82890  H 3.31901 -3.27656 0.44607  H 2.18853 -2.53970 1.61881  C 0.25895 3.60763 0.10470  H 0.90726 4.48679 0.12186  H -0.33635 3.57587 1.02863  H -0.41774 3.67020 -0.75968 | **A4I^LiOH^**  E(M06) = -614.735882 Hartrees.  ZPVE = 142.066 kcal mol^-1^  NImag = 0  H -1.98374 1.50263 -0.34465  C -1.11205 0.85523 -0.25965  C 1.14136 -0.79131 -0.03822  C -1.25316 -0.51235 -0.04498  C 0.16390 1.40660 -0.36554  C 1.28162 0.58061 -0.25336  C -0.13772 -1.34615 0.06701  H 2.27276 1.02342 -0.33720  C 0.33317 2.89242 -0.52645  H 1.25816 3.10296 -1.08357  H -0.49125 3.29737 -1.13236  C 0.37442 3.60827 0.82047  H 1.20720 3.23650 1.43217  H -0.55065 3.42764 1.38416  H 0.49534 4.69243 0.70120  O -0.27817 -2.67842 0.26897  O -2.44065 -1.18151 0.07184  O 2.16827 -1.66781 0.07807  C 3.46908 -1.15187 -0.02434  H 3.64490 -0.68467 -1.00579  H 4.14815 -1.99997 0.09553  H 3.67543 -0.41005 0.76277  C -3.62585 -0.43446 -0.03353  H -4.45066 -1.14116 0.08948  H -3.70913 0.05058 -1.01753  H -3.68810 0.33408 0.75121  H -1.23263 -2.85181 0.30505  **A4II^LiOH^**  E(M06) = -688.731116 Hartrees.  ZPVE = 130.330495 kcal mol^-1^  NImag = 0  C -2.66249 -0.94940 0.00939  C -1.25704 -0.47070 -0.04904  C 1.42343 0.31316 -0.16909  C -0.92500 0.88965 0.03500  C -0.24552 -1.42015 -0.18405  C 1.08791 -1.04219 -0.24698  C 0.40491 1.26993 -0.02298  H -1.70301 1.64097 0.14641  H -0.50632 -2.47395 -0.25097  O -2.92685 -2.13636 -0.05262  O 2.04877 -1.98964 -0.43534  O 0.87865 2.55024 0.03969  O 2.71342 0.69984 -0.24301  H 2.72283 1.67025 -0.18428  C 2.90077 -2.19976 0.67587  H 3.50658 -1.31179 0.89964  H 3.56117 -3.02951 0.40881  H 2.31693 -2.47620 1.56717  C -0.06200 3.59281 0.12509  H 0.50735 4.52540 0.13866  H -0.65789 3.52006 1.04674  H -0.73719 3.59240 -0.74318  C -3.76373 0.07527 0.15028  H -3.75051 0.79179 -0.68156  H -4.72549 -0.44398 0.16242  H -3.65382 0.65021 1.07944 |

Table S5**.** The Cartesian coordinates, total energy, zero-point vibrational energy and number of imaginary frequencies, of the optimized structures in Figure 5.

| **A1+ NaO*t*Bu**  **A1**  E(M06) = -1303.455988 Hartrees.  ZPVE = 144.0872275 kcal mol^-1^  NImag = 0  H -4.24575 -0.48267 -2.22194  C -3.82513 -0.23280 -1.24827  C -2.72580 0.32308 1.24251  C -2.88354 -1.10916 -0.69997  C -4.21007 0.91531 -0.57299  C -3.66296 1.18736 0.68568  C -2.31008 -0.82697 0.54299  H -3.98113 2.08114 1.22086  O -2.53828 -2.18191 -1.45511  O -2.12763 0.51272 2.44614  C -2.44191 -3.45375 -0.82211  H -1.43884 -3.62196 -0.41901  H -2.66564 -4.19318 -1.59871  H -3.18268 -3.54291 -0.01606  C -2.50269 1.64578 3.18635  H -2.27887 2.57774 2.64473  H -1.91809 1.61819 4.10952  H -3.57401 1.62970 3.43677  C -5.26435 1.82314 -1.14398  H -5.25442 1.75416 -2.24135  H -5.02978 2.86920 -0.89486  C -6.65519 1.47481 -0.62257  H -6.91892 0.44161 -0.88388  H -7.42125 2.14008 -1.04012  H -6.69355 1.55587 0.47193  O -1.37969 -1.66439 1.09778  C -0.04488 -1.18664 1.03781  H 0.53512 -1.79919 1.74092  C 0.57367 -1.32346 -0.34231  H -0.11806 -0.86224 -1.07392  C 1.89611 -0.59716 -0.38052  C 4.33316 0.77884 -0.33308  C 1.92640 0.76838 -0.68227  C 3.06977 -1.25358 -0.04118  C 4.29232 -0.57867 -0.01449  C 3.13643 1.44298 -0.64941  H 1.00045 1.28119 -0.94039  H 3.05828 -2.31262 0.20859  H 0.00279 -0.13766 1.36613  O 0.70604 -2.70678 -0.59218  H 1.08737 -2.81011 -1.47522  O 5.40205 -1.30924 0.27406  O 3.31950 2.76734 -0.93537  O 5.50930 1.45726 -0.36372  H 5.29502 2.37153 -0.61315  C 6.31552 -0.77506 1.21500  H 6.95992 -0.00828 0.77181  H 6.92536 -1.61637 1.55822  H 5.78464 -0.34116 2.07511  C 2.18926 3.51569 -1.31120  H 2.53767 4.53468 -1.49722  H 1.43582 3.53008 -0.51016  H 1.73181 3.11448 -2.22735  **NaO*t*Bu**  E(M06) = -395.194235 Hartrees.  ZPVE = 77.48505 kcal mol^-1^  NImag = 0  C 0.61085 0.03809 0.00000  C 1.14400 -0.68282 1.24705  H 0.76980 -1.71716 1.26116  H 0.77468 -0.17706 2.15108  H 2.24386 -0.71214 1.29097  C 1.14400 -0.68282 -1.24705  H 0.77468 -0.17706 -2.15108  H 0.76980 -1.71716 -1.26116  H 2.24386 -0.71214 -1.29097  C 1.14400 1.47831 0.00000  H 0.77250 2.00850 -0.88889  H 2.24397 1.52933 0.00000  H 0.77250 2.00850 0.88889  O -0.75406 0.03858 0.00000  Na -2.69003 -0.14062 0.00000 | **A2^NaO^*^t^*^Bu^**  E(M06) = -1698.709307 Hartrees.  ZPVE = 355.279205 kcal mol^-1^  NImag = 0  H 5.30833 -1.42780 0.50949  C 4.36309 -1.06351 0.11080  C 1.96600 -0.13222 -0.95379  C 3.15333 -1.53507 0.61695  C 4.37903 -0.12599 -0.92148  C 3.17944 0.32907 -1.46106  C 1.94328 -1.04113 0.10709  H 3.20121 1.04583 -2.27972  C 5.68870 0.32947 -1.50363  H 5.57124 1.33489 -1.93284  H 6.43950 0.41827 -0.70485  C 6.19312 -0.63097 -2.57667  H 5.46510 -0.71846 -3.39398  H 6.34287 -1.63651 -2.16177  H 7.14562 -0.29290 -3.00245  O 0.73569 -1.47907 0.58554  C 0.46664 -1.22951 1.97338  H 1.21006 -0.52736 2.37457  H 0.54162 -2.17047 2.53018  C -0.92323 -0.63592 2.13258  C -1.08589 0.67968 1.41210  C -1.33943 3.08023 0.00452  C -0.27520 1.76916 1.72795  C -2.05834 0.79727 0.41585  C -2.16974 1.99094 -0.28696  C -0.39316 2.96621 1.02955  H -2.68977 -0.06906 0.16810  O -1.06391 -0.51105 3.53623  H -1.89395 -0.04302 3.70017  Na -0.96573 -1.42764 -1.04618  O 3.03410 -2.45338 1.60257  O 0.74837 0.22756 -1.46216  C 0.68937 1.42948 -2.21315  H 1.16334 2.25415 -1.66375  H -0.37178 1.66142 -2.34621  H 1.16403 1.31043 -3.19710  C 4.21641 -2.96910 2.16843  H 3.90128 -3.68031 2.93539  H 4.81920 -2.17603 2.63418  H 4.82519 -3.49320 1.41758  H -1.66284 -1.35046 1.71731  O -1.41754 4.21162 -0.73862  O 0.47508 3.98423 1.30296  O -2.98864 -1.79536 -0.81188  H 0.47030 1.71070 2.52065  C -0.12574 5.15518 1.82551  H 0.69060 5.83775 2.07925  H -0.69857 4.92399 2.73653  H -0.78749 5.63483 1.09336  H -2.14317 4.06063 -1.37022  O -3.05343 2.22851 -1.30524  C -3.40513 1.12542 -2.13132  H -2.54504 0.83916 -2.76283  H -4.21888 1.46977 -2.77500  H -3.70705 0.23255 -1.56434  C -4.04403 -2.63685 -0.57669  C -4.82673 -2.89438 -1.87258  H -4.15776 -3.33653 -2.62545  H -5.68337 -3.57142 -1.72955  H -5.20448 -1.94260 -2.27564  C -4.99683 -2.01178 0.45394  H -5.86453 -2.64976 0.68280  H -4.45445 -1.82017 1.39273  H -5.37089 -1.04615 0.07987  C -3.54883 -3.98308 -0.02847  H -2.97610 -3.81330 0.89628  H -4.36393 -4.68934 0.19432  H -2.87689 -4.45551 -0.76147 | **TS1^NaO^*^t^*^Bu^**  E(M06) = -1698.675396 Hartrees.  ZPVE = 351.0981725 kcal mol^-1^  NImag = 1  H 5.66168 0.72152 -1.45668  C 4.83294 0.34912 -0.85687  C 2.75051 -0.61773 0.69599  C 3.59690 0.99406 -0.88222  C 5.03501 -0.77283 -0.05393  C 3.99153 -1.24861 0.73381  C 2.52667 0.49739 -0.11830  H 4.15437 -2.11291 1.37608  C 6.39380 -1.41343 0.01810  H 6.28688 -2.48588 0.23750  H 6.88821 -1.34497 -0.96207  C 7.27170 -0.75855 1.08056  H 6.80695 -0.83781 2.07228  H 7.40919 0.30992 0.86795  H 8.26263 -1.22658 1.12974  O 1.30399 1.09577 -0.09669  C 0.56614 1.15929 -1.37798  H 0.86314 0.24378 -1.92640  H 0.92373 2.03162 -1.93280  C -0.90295 1.30053 -1.11867  C -1.65590 0.05379 -1.00140  C -3.21543 -2.25445 -0.39490  C -1.07914 -1.11926 -0.47235  C -3.03666 0.00902 -1.28991  C -3.78526 -1.11601 -0.98802  C -1.84498 -2.24640 -0.16409  H -3.48571 0.87729 -1.76385  O -1.50745 2.20284 -2.05416  H -1.58829 3.05555 -1.60712  Na -0.16070 0.47460 1.59216  O 3.32993 2.10264 -1.61016  O 1.68612 -1.02698 1.46396  C 1.64125 -2.39651 1.83444  H 1.89766 -3.03393 0.97768  H 0.60873 -2.61314 2.12446  H 2.32050 -2.60589 2.67185  C 4.35658 2.62552 -2.41669  H 3.93529 3.50027 -2.91784  H 4.68600 1.89861 -3.17397  H 5.22363 2.93691 -1.81537  H -1.15170 1.98019 0.26526  O -3.99188 -3.32354 -0.04826  O -1.20584 -3.31111 0.42163  O -1.24974 2.38057 1.31744 |
| --- | --- | --- |
| **A3^NaO^*^t^*^Bu^**  **(Phenolate)**  E(M06) = -776.456172 Hartrees.  ZPVE = 134.50086 kcal mol^-1^  NImag = 0  H 2.23307 1.82621 -0.14401  C 1.49784 1.01878 -0.13890  C -0.40780 -0.93954 -0.10504  C 0.17013 1.36980 0.04202  C 1.90932 -0.31073 -0.29444  C 0.92389 -1.29157 -0.28012  C -0.87016 0.39180 0.07701  H 1.17678 -2.34715 -0.40244  O -0.11785 2.69381 0.25184  O -1.37575 -1.96766 -0.07369  C -1.03419 3.24996 -0.66955  H -0.64372 3.17477 -1.69793  H -1.13933 4.30846 -0.40813  H -2.00756 2.74791 -0.61194  C -1.94630 -2.16540 -1.35853  H -2.69997 -2.95825 -1.27308  H -1.17396 -2.48120 -2.07378  H -2.41414 -1.23740 -1.71876  C 3.36687 -0.65741 -0.41772  H 3.47524 -1.63408 -0.91394  H 3.87240 0.07429 -1.06709  C 4.06930 -0.69513 0.93638  H 5.13620 -0.93629 0.83919  H 3.60662 -1.44620 1.59099  H 3.98390 0.27594 1.44256  O -2.11795 0.68075 0.29268  Na -2.78059 -0.94959 1.44773  **(Hydroxylvinyl)**  E(M06) = -688.699461 Hartrees.  ZPVE = 130.55765 kcal mol^-1^  NImag = 0  C -3.64749 0.24098 0.73692  H -4.70904 0.01179 0.72279  C -2.78272 -0.51950 0.05804  C -1.33123 -0.27564 -0.01673  C 1.43669 0.16770 -0.15835  C -0.82810 1.03261 0.01100  C -0.44071 -1.34387 -0.11571  C 0.93511 -1.13293 -0.18530  C 0.53882 1.24297 -0.04910  H -1.52494 1.86604 0.04841  H -0.78808 -2.37562 -0.09955  H -3.30189 1.06270 1.35580  O -3.23980 -1.63418 -0.58558  H -2.60603 -1.87192 -1.27771  O 1.75300 -2.21381 -0.32125  O 1.15929 2.45885 -0.04925  O 2.76698 0.39772 -0.25642  H 2.88816 1.36156 -0.22825  C 2.66801 -2.41915 0.74056  H 3.41445 -1.61754 0.79622  H 3.16903 -3.36995 0.53737  H 2.13705 -2.49011 1.70191  C 0.34603 3.60715 -0.00232  H 1.01976 4.46727 -0.01224  H -0.25893 3.62898 0.91571  H -0.32190 3.65630 -0.87452 | **TS2 ^NaO^*^t^*^Bu^**  E(M06) = -688.623968 Hartrees.  ZPVE = 126881755 kcal mol^-1^  NImag = 1  C -3.81814 0.19643 0.34299  H -4.46995 0.37193 -0.51953  C -2.70182 -0.62330 0.00741  C -1.28261 -0.33472 -0.04414  C 1.44732 0.20686 -0.17227  C -0.83659 0.99561 0.02050  C -0.36209 -1.37894 -0.15801  C 0.99896 -1.11745 -0.23042  C 0.51859 1.25554 -0.03512  H -1.56870 1.79610 0.09502  H -0.70603 -2.40976 -0.19391  H -3.70639 1.08031 0.97478  O -3.14393 -1.82021 -0.21625  H -4.13854 -1.25909 0.29052  O 1.87134 -2.14840 -0.39767  O 1.10602 2.48607 0.00703  O 2.76302 0.48052 -0.25270  H 2.85658 1.44771 -0.20266  C 2.75296 -2.37310 0.68897  H 3.44754 -1.53512 0.82890  H 3.31901 -3.27656 0.44607  H 2.18853 -2.53970 1.61881  C 0.25895 3.60763 0.10470  H 0.90726 4.48679 0.12186  H -0.33635 3.57587 1.02863  H -0.41774 3.67020 -0.75968 | **A4I ^NaO^*^t^*^Bu^**  E(M06) = -614.735882 Hartrees.  ZPVE = 142.066 kcal mol^-1^  NImag = 0  H -1.98374 1.50263 -0.34465  C -1.11205 0.85523 -0.25965  C 1.14136 -0.79131 -0.03822  C -1.25316 -0.51235 -0.04498  C 0.16390 1.40660 -0.36554  C 1.28162 0.58061 -0.25336  C -0.13772 -1.34615 0.06701  H 2.27276 1.02342 -0.33720  C 0.33317 2.89242 -0.52645  H 1.25816 3.10296 -1.08357  H -0.49125 3.29737 -1.13236  C 0.37442 3.60827 0.82047  H 1.20720 3.23650 1.43217  H -0.55065 3.42764 1.38416  H 0.49534 4.69243 0.70120  O -0.27817 -2.67842 0.26897  O -2.44065 -1.18151 0.07184  O 2.16827 -1.66781 0.07807  C 3.46908 -1.15187 -0.02434  H 3.64490 -0.68467 -1.00579  H 4.14815 -1.99997 0.09553  H 3.67543 -0.41005 0.76277  C -3.62585 -0.43446 -0.03353  H -4.45066 -1.14116 0.08948  H -3.70913 0.05058 -1.01753  H -3.68810 0.33408 0.75121  H -1.23263 -2.85181 0.30505  **A4II ^NaO^*^t^*^Bu^**  E(M06) = -688.731116 Hartrees.  ZPVE = 130.330495 kcal mol^-1^  NImag = 0  C -2.66249 -0.94940 0.00939  C -1.25704 -0.47070 -0.04904  C 1.42343 0.31316 -0.16909  C -0.92500 0.88965 0.03500  C -0.24552 -1.42015 -0.18405  C 1.08791 -1.04219 -0.24698  C 0.40491 1.26993 -0.02298  H -1.70301 1.64097 0.14641  H -0.50632 -2.47395 -0.25097  O -2.92685 -2.13636 -0.05262  O 2.04877 -1.98964 -0.43534  O 0.87865 2.55024 0.03969  O 2.71342 0.69984 -0.24301  H 2.72283 1.67025 -0.18428  C 2.90077 -2.19976 0.67587  H 3.50658 -1.31179 0.89964  H 3.56117 -3.02951 0.40881  H 2.31693 -2.47620 1.56717  C -0.06200 3.59281 0.12509  H 0.50735 4.52540 0.13866  H -0.65789 3.52006 1.04674  H -0.73719 3.59240 -0.74318  C -3.76373 0.07527 0.15028  H -3.75051 0.79179 -0.68156  H -4.72549 -0.44398 0.16242  H -3.65382 0.65021 1.07944 |

Table S6**.** The Cartesian coordinates, total energy, zero-point vibrational energy and number of imaginary frequencies, of the optimized structures in Figure 6.

| **B1+NaOH**  **B1**  E(M06) = -1417.925525 Hartrees.  ZPVE = 296.249025 kcal mol^-1^  NImag = 0  C 3.17374 0.48642 0.41406  C 2.45920 -1.80360 -0.98787  C 1.83688 0.28288 0.07290  C 4.14677 -0.44612 0.06077  C 3.79795 -1.58936 -0.64815  C 1.47614 -0.88241 -0.63076  H 5.18108 -0.24632 0.34031  H 2.19147 -2.70558 -1.53611  O 0.87978 1.17794 0.46988  C 0.36469 2.04214 -0.53747  H 0.28966 1.52048 -1.50594  C -1.05184 2.44979 -0.10415  H -0.94313 2.97971 0.86755  C -1.91318 1.23384 0.10602  C -3.34162 -1.14859 0.43962  C -2.52661 0.62438 -0.97958  C -2.03409 0.66779 1.37528  C -2.74459 -0.51175 1.53579  C -3.23627 -0.56157 -0.82078  H -2.44751 1.05747 -1.97436  H -1.54393 1.14546 2.22156  O -1.64687 3.27131 -1.07457  H -0.96067 3.87078 -1.41749  C 1.32453 3.21712 -0.65049  H 1.34873 3.73995 0.32379  H 2.33920 2.83768 -0.84523  O 0.88361 4.07169 -1.69370  H 1.46132 4.84425 -1.72835  O 3.56356 1.63049 1.05873  O 0.16380 -1.00431 -0.91968  C 3.13807 1.71997 2.40536  H 2.04366 1.76941 2.47948  H 3.57453 2.63759 2.81233  H 3.50298 0.85945 2.98693  C -0.25332 -2.12969 -1.65936  H 0.25276 -2.17636 -2.63522  H -1.33051 -2.01679 -1.81366  H -0.05897 -3.06256 -1.10952  C 4.83307 -2.62803 -0.98091  H 5.81180 -2.14520 -1.11612  H 4.58611 -3.10675 -1.94010  C 4.93311 -3.69064 0.10962  H 5.20629 -3.23502 1.07061  H 5.68552 -4.45094 -0.13395  H 3.96860 -4.19679 0.24929  O -3.74010 -1.18332 -1.93230  O -2.92991 -1.17941 2.71742  O -4.00124 -2.32449 0.60455  H -3.94745 -2.53938 1.55049  C -5.15114 -1.28671 -1.97044  H -5.53158 -1.94356 -1.17773  H -5.40806 -1.70858 -2.94690  H -5.61506 -0.29336 -1.87374  C -2.32106 -0.64211 3.86414  H -1.22829 -0.58921 3.74847  H -2.56592 -1.31174 4.69278  H -2.70607 0.36430 4.08638  **NaOH**  E(M06) = -238.055709 Hartrees.  ZPVE = 6.7224075 kcal mol^-1^  NImag = 0  O 0.02821 1.03311 0.00000  H -0.53591 1.80813 0.00000  Na 0.02821 -0.91573 0.00000 | **B2**  E(M06) = -1656.042904 Hartrees.  ZPVE = 305.5680275 kcal mol^-1^  NImag = 0  C 2.97839 0.67470 0.72095  C 3.10107 -1.55738 -0.98383  C 1.86652 0.30202 -0.03655  C 4.17020 -0.03740 0.55736  C 4.24405 -1.13114 -0.29579  C 1.91431 -0.85303 -0.83351  H 5.03745 0.28135 1.13505  H 3.15232 -2.44494 -1.61308  O 0.68149 0.99874 0.06374  C 0.35585 1.83512 -1.04556  H 0.39929 1.25558 -1.98391  C -1.09118 2.32294 -0.85031  H -1.07444 3.06807 -0.02677  C -2.00202 1.19545 -0.44660  C -3.52048 -1.02319 0.33691  C -2.36867 0.22998 -1.37929  C -2.41041 1.06654 0.88354  C -3.16957 -0.03318 1.27409  C -3.11743 -0.88251 -0.99548  H -2.06602 0.31830 -2.42118  H -2.07865 1.79212 1.62471  O -1.56515 2.88588 -2.04609  H -0.88188 3.50755 -2.35313  C 1.34912 2.98561 -1.09034  H 1.29591 3.52805 -0.12968  H 2.37220 2.59108 -1.19641  O 1.00628 3.81827 -2.18434  H 1.57216 4.60010 -2.17168  O 2.97656 1.73381 1.55296  O 0.71135 -1.20199 -1.38284  C 1.86835 1.90400 2.45456  H 1.33875 0.95506 2.67267  H 1.14530 2.62227 2.04122  H 2.30057 2.32336 3.36950  C 0.67061 -2.28562 -2.28858  H 1.31846 -2.09742 -3.15566  H -0.37101 -2.36397 -2.61368  H 0.97619 -3.22418 -1.80396  C 5.51758 -1.92420 -0.40048  H 6.37857 -1.25937 -0.24148  H 5.62086 -2.33275 -1.41671  C 5.55507 -3.06183 0.61575  H 5.46930 -2.67047 1.63778  H 6.48813 -3.63427 0.54523  H 4.71754 -3.75447 0.45839  O -3.36638 -1.85324 -1.92361  O -3.60711 -0.29268 2.52799  O -4.21048 -2.11619 0.74459  H -4.34740 -2.00238 1.70266  C -4.73937 -2.09113 -2.19066  H -5.25543 -2.50205 -1.31463  H -4.77572 -2.81463 -3.01001  H -5.23613 -1.16186 -2.50673  C -2.92327 0.35309 3.60985  H -1.83125 0.15277 3.53099  H -3.33963 -0.09092 4.51841  H -3.15300 1.42876 3.61012  O -0.08973 -0.57953 2.89670  H 0.22988 -1.15306 3.60060  Na -0.47176 -0.90867 0.89296 | **TSBI**  E(M06) = -1656.002679 Hartrees.  ZPVE = 301.05191 kcal mol^-1^  NImag = 1  C -3.54467 -1.06048 -0.05641  C -3.58298 1.73031 -0.17467  C -2.35326 -0.34656 0.11384  C -4.72668 -0.38011 -0.34678  C -4.75286 1.00736 -0.42724  C -2.40175 1.05684 0.10978  H -5.63157 -0.96806 -0.49977  H -3.61510 2.81911 -0.18815  O -1.17525 -0.97683 0.39655  C -0.27904 -1.32955 -0.72451  H -0.23690 -0.43567 -1.37433  C 1.06043 -1.70070 -0.12162  H 0.86656 -1.83010 1.23293  C 2.10909 -0.65711 -0.20594  C 4.14563 1.31458 0.02948  C 1.82251 0.71574 -0.26811  C 3.45998 -1.02530 -0.05334  C 4.44544 -0.05865 0.04681  C 2.81938 1.68778 -0.14331  H 0.81437 1.07730 -0.46762  H 3.69413 -2.08584 -0.01433  O 1.52621 -2.99157 -0.48968  H 1.55847 -3.01288 -1.46221  C -0.89075 -2.44681 -1.54392  H -0.89765 -3.37544 -0.95267  H -1.92586 -2.20040 -1.82177  O -0.07111 -2.56307 -2.69910  H -0.41248 -3.29091 -3.23350  O -3.58056 -2.41879 -0.00283  O -1.22315 1.67716 0.43329  C -3.13181 -3.00689 1.21647  H -2.06510 -2.82643 1.39748  H -3.31768 -4.08009 1.11861  H -3.71734 -2.61570 2.06148  C -1.08294 3.05245 0.12015  H -1.39162 3.24497 -0.91610  H -0.01738 3.28409 0.22860  H -1.67776 3.67914 0.79958  C -6.04589 1.73254 -0.68030  H -6.69079 1.12063 -1.32728  H -5.84810 2.66504 -1.22926  C -6.77884 2.04897 0.62012  H -7.00671 1.12722 1.17140  H -7.72211 2.57674 0.43320  H -6.16024 2.67815 1.27414  O 2.43253 3.00613 -0.14947  O 5.78209 -0.30568 0.20040  O 5.13237 2.24213 0.19043  H 5.95845 1.74177 0.29315  C 3.00644 3.78988 -1.18139  H 4.09084 3.89114 -1.05428  H 2.53729 4.77709 -1.12322  H 2.79577 3.34641 -2.16661  C 6.19075 -1.64925 0.28472  H 5.72704 -2.15470 1.14424  H 7.27643 -1.63698 0.41234  H 5.93747 -2.20173 -0.63147  O 0.71749 -1.75171 2.49573  H 1.52223 -2.14171 2.85638  Na 0.20922 0.23959 1.94122 |
| --- | --- | --- |
| **TSBII**  E(M06) = -1656.021769 Hartrees.  ZPVE = 303.889465 kcal mol^-1^  NImag = 1  C 2.49117 0.38268 -0.25941  C 1.23775 -1.98387 0.45160  C 1.73260 0.36748 0.92686  C 2.56969 -0.72793 -1.09189  C 1.91876 -1.91060 -0.76703  C 1.15562 -0.87369 1.27972  H 3.11683 -0.62813 -2.02935  H 0.72391 -2.91153 0.70322  O 1.51394 1.46766 1.64306  C 0.53032 2.78412 0.63334  H 1.08168 2.57038 -0.27719  C -0.91607 2.46475 0.55737  H -1.43830 2.53902 1.54763  C -1.27553 1.12347 -0.03040  C -1.70781 -1.47619 -0.97825  C -0.77255 0.72681 -1.26343  C -2.06755 0.23701 0.70246  C -2.28286 -1.04830 0.22158  C -0.97245 -0.56431 -1.74388  H -0.18340 1.41377 -1.86906  H -2.51751 0.56645 1.64100  O -1.03559 3.57769 -0.22366  H -0.78455 4.66622 1.08123  C 0.94535 4.01171 1.41841  H 1.53450 3.70908 2.29524  H 1.59699 4.64022 0.79101  O -0.16882 4.75298 1.87025  H -0.67779 3.90261 3.26859  O 3.07773 1.54831 -0.69154  O 0.44921 -0.87393 2.47612  C 4.05749 2.07655 0.18018  H 3.62590 2.35168 1.15206  H 4.46644 2.96642 -0.30936  H 4.86856 1.34925 0.33969  C -0.12975 -2.09436 2.87596  H -0.90834 -2.42390 2.17070  H -0.58316 -1.92189 3.85864  H 0.63021 -2.88300 2.97082  C 1.94356 -3.09865 -1.68743  H 2.14081 -2.75420 -2.71322  H 0.94259 -3.55891 -1.70845  C 2.98488 -4.13491 -1.27832  H 3.99104 -3.69458 -1.27750  H 2.99074 -4.99691 -1.95789  H 2.79129 -4.50686 -0.26263  O -0.35960 -0.90286 -2.90902  O -2.99289 -2.03400 0.86962  O -1.84205 -2.77265 -1.37111  H -2.43075 -3.19701 -0.72542  C -1.09834 -1.64239 -3.86310  H -1.09884 -2.71520 -3.63813  H -0.61105 -1.46905 -4.82784  H -2.13833 -1.28948 -3.91809  C -3.79383 -1.64297 1.95583  H -3.18261 -1.30561 2.80886  H -4.36735 -2.52321 2.25842  H -4.48311 -0.83428 1.67261  O -0.76907 3.24300 4.01023  H -0.69050 3.75461 4.82289  Na -0.02888 1.29370 3.18986 | **B3I**  **(Phenolate)**  E(M06) = -776.456172 Hartrees.  ZPVE = 134.50086 kcal mol^-1^  NImag = 0  H 2.23307 1.82621 -0.14401  C 1.49784 1.01878 -0.13890  C -0.40780 -0.93954 -0.10504  C 0.17013 1.36980 0.04202  C 1.90932 -0.31073 -0.29444  C 0.92389 -1.29157 -0.28012  C -0.87016 0.39180 0.07701  H 1.17678 -2.34715 -0.40244  O -0.11785 2.69381 0.25184  O -1.37575 -1.96766 -0.07369  C -1.03419 3.24996 -0.66955  H -0.64372 3.17477 -1.69793  H -1.13933 4.30846 -0.40813  H -2.00756 2.74791 -0.61194  C -1.94630 -2.16540 -1.35853  H -2.69997 -2.95825 -1.27308  H -1.17396 -2.48120 -2.07378  H -2.41414 -1.23740 -1.71876  C 3.36687 -0.65741 -0.41772  H 3.47524 -1.63408 -0.91394  H 3.87240 0.07429 -1.06709  C 4.06930 -0.69513 0.93638  H 5.13620 -0.93629 0.83919  H 3.60662 -1.44620 1.59099  H 3.98390 0.27594 1.44256  O -2.11795 0.68075 0.29268  Na -2.78059 -0.94959 1.44773  **(HydroxylPropenyl)**  E(M06) = -803.155658 Hartrees.  ZPVE = 152.0627025 kcal mol^-1^  NImag = 0  C -3.00581 0.84705 -0.15825  H -2.62929 1.76331 -0.60519  C -2.14330 -0.14412 0.15132  C -0.68391 -0.06670 -0.00949  C 2.10364 0.02340 -0.36170  C 0.07543 -1.23937 -0.06390  C -0.02568 1.16384 -0.10166  C 1.34491 1.19483 -0.27708  C 1.45286 -1.21045 -0.24449  H -0.40659 -2.20974 0.01915  H -0.56735 2.10545 -0.02739  O -2.55975 -1.33810 0.63376  H -3.52913 -1.38892 0.49262  C -4.47427 0.72024 0.09067  H -4.72114 0.78525 1.16109  H -5.02873 1.52650 -0.41275  O -4.99408 -0.55898 -0.30094  H -4.77508 -0.67062 -1.23869  O 2.12489 -2.39100 -0.35281  O 2.04598 2.37855 -0.39710  O 3.43976 0.09635 -0.56093  H 3.62900 1.03753 -0.72687  C 3.12431 -2.61186 0.62565  H 3.95601 -1.90314 0.52598  H 3.49296 -3.62953 0.46800  H 2.69985 -2.53482 1.63827  C 2.28963 3.00488 0.85116  H 1.34815 3.26278 1.35664  H 2.85233 3.92043 0.64625  H 2.87780 2.35054 1.51383 | **B3II**  **(Phenolate)**  E(M06) = -776.456172 Hartrees.  ZPVE = 134.50086 kcal mol^-1^  NImag = 0  H 2.23307 1.82621 -0.14401  C 1.49784 1.01878 -0.13890  C -0.40780 -0.93954 -0.10504  C 0.17013 1.36980 0.04202  C 1.90932 -0.31073 -0.29444  C 0.92389 -1.29157 -0.28012  C -0.87016 0.39180 0.07701  H 1.17678 -2.34715 -0.40244  O -0.11785 2.69381 0.25184  O -1.37575 -1.96766 -0.07369  C -1.03419 3.24996 -0.66955  H -0.64372 3.17477 -1.69793  H -1.13933 4.30846 -0.40813  H -2.00756 2.74791 -0.61194  C -1.94630 -2.16540 -1.35853  H -2.69997 -2.95825 -1.27308  H -1.17396 -2.48120 -2.07378  H -2.41414 -1.23740 -1.71876  C 3.36687 -0.65741 -0.41772  H 3.47524 -1.63408 -0.91394  H 3.87240 0.07429 -1.06709  C 4.06930 -0.69513 0.93638  H 5.13620 -0.93629 0.83919  H 3.60662 -1.44620 1.59099  H 3.98390 0.27594 1.44256  O -2.11795 0.68075 0.29268  Na -2.78059 -0.94959 1.44773  **(Epoxide prdt)**  E(M06) = -803.155658 Hartrees.  ZPVE = 152.0627025 kcal mol^-1^  NImag = 0  C 2.99948 -0.57752 0.57410  H 2.55545 -1.18349 1.37119  C 2.08622 0.12868 -0.32816  H 2.47536 1.03717 -0.79845  C 0.61396 0.06224 -0.17868  C -2.15933 -0.02430 0.17772  C -0.03841 -1.15376 -0.02735  C -0.12127 1.25129 -0.18039  C -1.49516 1.19883 -0.00068  C -1.42121 -1.20936 0.14605  H 0.51411 -2.09026 -0.06560  H 0.38961 2.20240 -0.31887  O 2.75843 -1.06339 -0.73871  H 4.67881 -0.03551 -1.08917  C 4.41294 -0.10225 0.79694  H 4.44400 0.61859 1.62463  H 5.03578 -0.96622 1.09472  O 4.92502 0.53360 -0.34138  O -1.97989 -2.43445 0.33082  O -2.33432 2.27693 0.03988  O -3.50034 -0.04285 0.38598  H -3.79863 0.88150 0.36224  C -3.13062 -2.75317 -0.43102  H -4.04080 -2.32322 0.00111  H -3.20388 -3.84501 -0.43142  H -3.02552 -2.39977 -1.46703  C -1.76234 3.55459 -0.09823  H -1.02222 3.74705 0.69227  H -2.57981 4.27449 -0.01072  H -1.27904 3.67198 -1.07940 |
